# A Novel Function for the Hox Gene *Abd-B* in the Male Accessory Gland Regulates the Long-Term Female Post-Mating Response in *Drosophila*

**DOI:** 10.1371/journal.pgen.1003395

**Published:** 2013-03-28

**Authors:** Dragan Gligorov, Jessica L. Sitnik, Robert K. Maeda, Mariana F. Wolfner, François Karch

**Affiliations:** 1 Department of Genetics and Evolution and NCCR Frontiers in Genetics, University of Geneva, Geneva, Switzerland; 2 Department of Molecular Biology and Genetics, Cornell University, Ithaca, New York, United States of America; New York University, United States of America

## Abstract

In insects, products of the male reproductive tract are essential for initiating and maintaining the female post-mating response (PMR). The PMR includes changes in egg laying, receptivity to courting males, and sperm storage. In *Drosophila*, previous studies have determined that the main cells of the male accessory gland produce some of the products required for these processes. However, nothing was known about the contribution of the gland's other secretory cell type, the secondary cells. In the course of investigating the late functions of the homeotic gene, *Abdominal-B (Abd-B)*, we discovered that *Abd-B* is specifically expressed in the secondary cells of the *Drosophila* male accessory gland. Using an *Abd-B* BAC reporter coupled with a collection of genetic deletions, we discovered an enhancer from the *iab-6* regulatory domain that is responsible for *Abd-B* expression in these cells and that apparently works independently from the segmentally regulated chromatin domains of the bithorax complex. Removal of this enhancer results in visible morphological defects in the secondary cells. We determined that mates of *iab-6* mutant males show defects in long-term egg laying and suppression of receptivity, and that products of the secondary cells are influential during sperm competition. Many of these phenotypes seem to be caused by a defect in the storage and gradual release of sex peptide in female mates of *iab-6* mutant males. We also found that *Abd-B* expression in the secondary cells contributes to glycosylation of at least three accessory gland proteins: ovulin (Acp26Aa), CG1656, and CG1652. Our results demonstrate that long-term post-mating changes observed in mated females are not solely induced by main cell secretions, as previously believed, but that secondary cells also play an important role in male fertility by extending the female PMR. Overall, these discoveries provide new insights into how these two cell types cooperate to produce and maintain a robust female PMR.

## Author Summary

Similar to the prostate gland and seminal vesicle in mammals, the Drosophila male accessory gland produces seminal fluid proteins that are critical for individual male reproductive success. In Drosophila, many of these proteins function to induce a suite of long-lasting physiological changes in mated females, like increased egg laying and decreased receptivity to secondary courtships. While investigating the *cis*-regulatory region of the Hox gene, *Abd-B*, we found that *Abd-B* is specifically expressed in a mostly uncharacterized cell-type of the accessory gland, called the secondary cells. Using regulatory mutants of *Abd-B*, we were able to perturb the function of the secondary cells to show that the secondary cells play a critical role in extending the duration of the post-mating response in females. The induced physiological changes in the female result in a genetic advantage for the genes of the copulating male in populating the next generation. Interestingly, the function of the *Abd-B* class genes in seminal protein producing tissues seems to be an ancient and conserved function, as the orthologues of *Abd-B* in mammals, the *hox9-13* class of genes, are expressed in the mammalian prostate and seminal vesicle.

## Introduction

The homeotic transcription factor *Abdominal-B* (*Abd-B*) specifies the identity of the four most-posterior abdominal segments of the fly (the 5^th^ through 8^th^ abdominal segments), as well as the genital and anal structures [1]–[3]. Each of these segments is specified by a particular pattern and level of Abd-B protein expression in the early embryo. Four segment-specific *cis*-regulatory domains (*iab-5* through *iab-8*) spanning >90 kb of DNA have been shown to control the expression pattern of *Abd-B*, where each domain is predominantly responsible for controlling the *Abd-B* expression pattern in one particular segment [4]–[6] (for a review see [7]).

Extensive study has been devoted to exploring how the segment-specific expression pattern of *Abd-B* is achieved. Due to the striking cuticular transformations elicited by *Abd-B* mutations, genetic and transgenic analyses have been able to discover numerous enhancers, silencers and insulators that direct *Abd-B* expression in the ectoderm [8]–[16]. However, much less is known about the role of *Abd-B* in non-ectodermally derived tissues during later stages of development. Here, we use a 111 kb BAC-based reporter construct to identify new locations of *Abd-B* expression in the adult fly. We find that *Abd-B* is strongly expressed in the accessory gland (AG), a secretory tissue of the adult male reproductive tract that has important reproductive functions.

The AG synthesizes seminal proteins that are essential for male fertility. These >180 accessory gland proteins (“Acps”) are transferred to females during mating and cause post-mating changes in the females known collectively as the post-mating response (PMR). The PMR includes increased rates of egg-laying and ovulation, sperm storage, decreased receptivity to courting males, as well as changes in longevity, feeding, and sleep patterns (reviewed in [17], [18]). The PMR is divided into two phases. The short term response (STR) refers to changes in the above behaviors during the first ∼24 hours post-mating. It requires Acps, but not the receipt of sperm. Persistence of the PMR after 24 hr (and for up to ∼10 days) is known as the long-term response (LTR). The LTR requires Acps and stored sperm [19]–[22]. Many of the roles of Acps were initially discovered by experiments in which whole AG extracts or purified Acps were injected into unmated females [23]–[25], or by whole-tissue ablation in males [26].

Each lobe of the AG is composed of a monolayer of approximately 1000 secretory cells comprised of two morphologically distinct cell types. Roughly 96% of these cells are flat, polygonally shaped “main cells”. The remaining 4% of the cells are large, spherical, vacuole filled “secondary cells ”; these are dispersed among the main cells at the distal tip of the gland. Enhancer trapping and other studies have shown that, in addition to their morphological differences, these two secretory cell types are biochemically distinct [27]–[29]. Ablation of the main cells only [19] showed that products of these cells are essential for the PMR. These products include ovulin (Acp26Aa), an Acp that acts in the STR to stimulate ovulation [30], [31], and the sex peptide (SP, Acp70A), which is the ultimate regulator of most other PMR effects [22], [32]–[35]. SP binds to sperm within the mated female, and its active portion is gradually released from the sperm [22]. This binding and release allows SP to affect the female for as long as she contains stored sperm. A network of five other Acps is necessary for SP to bind to sperm and enter storage. The predicted protease CG10586 (Seminase) [36] appears to be necessary for both STR and LTR related events, while the predicted protease CG9997, the predicted cysteine-rich secretory protein (CRISP) CG17575, and the predicted lectins CG1656/1652 appear to be LTR specific [37]–[40]. The cellular source of each of these proteins is currently unknown.

In spite of the detailed characterization of the main cells and several specific Acps, the role of the secondary cells has remained mysterious. No PMR-associated Acps were known to be expressed exclusively in the secondary cells, and no tools have been available to specifically target those cells. Here, we identified the secondary cells of the male AG as a novel location of *Abd-B* expression in the adult fly. By screening an extensive collection of *cis*-regulatory deletions [6], [41], [42], we discovered a 2.8 kb enhancer from the *iab-6 cis*-regulatory domain, whose removal completely abolishes *Abd-B* expression in the secondary cells. Loss of *Abd-B* expression in the secondary cells causes those cells to develop aberrantly. Moreover, these mutant males provide their mates with substances that initiate the PMR, but are insufficient to maintain it. Our results indicate that *Abd-B* expression in the secondary cells is essential for their proper development and for the production of proteins important for long-term changes in female post-mating responses.

## Results

### Creation of Gal4 reporter BAC for *Abdominal B*

**Figure 1:**
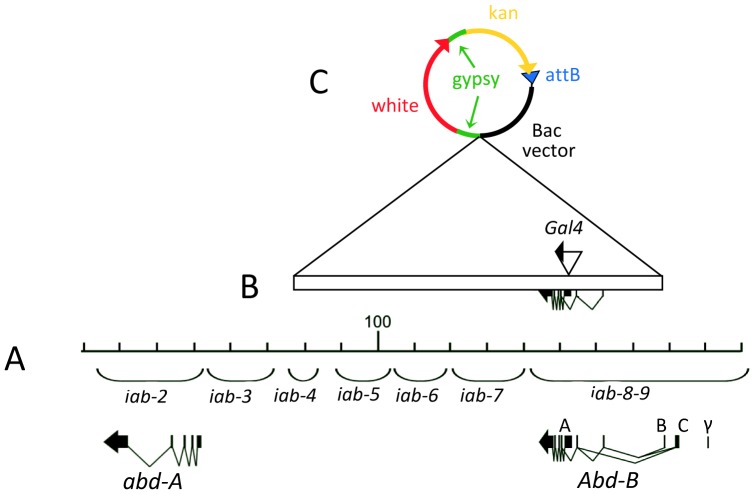
Extend of DNA contains in the *Abd-B* BAC. A) Molecular map of the abdominal region of the Bithorax complex numbered in kb according to [88] (Genbank U31961). The *abd-A* and *Abd-B* transcription units are drawn below the DNA line along with the extent of the segment-specific *iab cis*-regulatory domains *iab-2* through *iab-9*. B) The rectangle depicts the extent of the BAC used in this study. Note that it lacks the B,C and γ promoters specific for the *Abd-Br* form. The Gal-4 coding sequence was inserted within the 5′UTR of the *Abd-Bm* form. C) The structure of the vector sequences used to propagate the BAC and to select the integration within the *Drosophila* genome. Note the presence of two gypsy insulator sequences flanking the mini-*white* sequences to prevent possible position effect on *white* expression (see Materials and Methods for further details).

In order to discover new tissues in which the *Abd-B* gene functions, we undertook the creation of a transgenic reporter that accurately reproduces the *Abd-B* expression pattern throughout development. Previous studies indicated that the *Abd-B* gene is expressed as two isoforms, the Abd-B *m* and *r* forms, and that the expression of these two isoforms requires separate elements located within a large *cis*-regulatory region spanning >90 kb of DNA [43]. As the *Abd-Br* isoform is thought to be primarily involved in the formation of the external genitalia [44], we decided to concentrate our study on the *Abd-Bm* isoform, which is involved in determining segment identity. BACR24L18 is a BAC of ∼172 kb that contains the *Abd-B*, and much of the *abd-A* region of the Bithorax complex (BX-C). By recombineering, we reduced BAC24L18 to contain mostly the *iab-5* to *iab-8* domains required for *Abd-Bm* expression (removing many of the *Abd-Br* alternative promoters and its regulatory elements) and the *Abd-Bm* coding sequence (Figure 1B). A ΦC31 AttB integration sequence and a *white* integration marker were also added during the reduction step (Figure 1B and 1C).

We first tested if expression derived from the sequences on this BAC were sufficient to rescue *Abd-B* mutant phenotypes. We integrated the *Abd-B* BAC into the 51C landing platform [45] and tested for complementation of two large deletions affecting *Abd-B* activity. We found that the presence of a copy of the BAC on the second chromosome rescues the mutant phenotypes of *iab*-6,7^IH^ and *iab*-5,6^J82^ [6] (Figure S1; See Text S1). Because the sequences preserved on the BAC seemed to drive appropriate *Abd-Bm* expression, we proceeded to modify the BAC by recombineering to replace the first codon of the first exon of *Abd-B* with the sequence encoding the Gal4 transcription factor. As this sequence also adds a stop codon, the expression of *Abd-B* from the BAC should be eliminated, but any sequences that might be used in *Abd-B* gene regulation will be preserved to drive reporter gene expression. The final BAC used in the experiments was 111 kb (Figure 1). It was integrated into the 51C landing platform.

**Figure 2:**
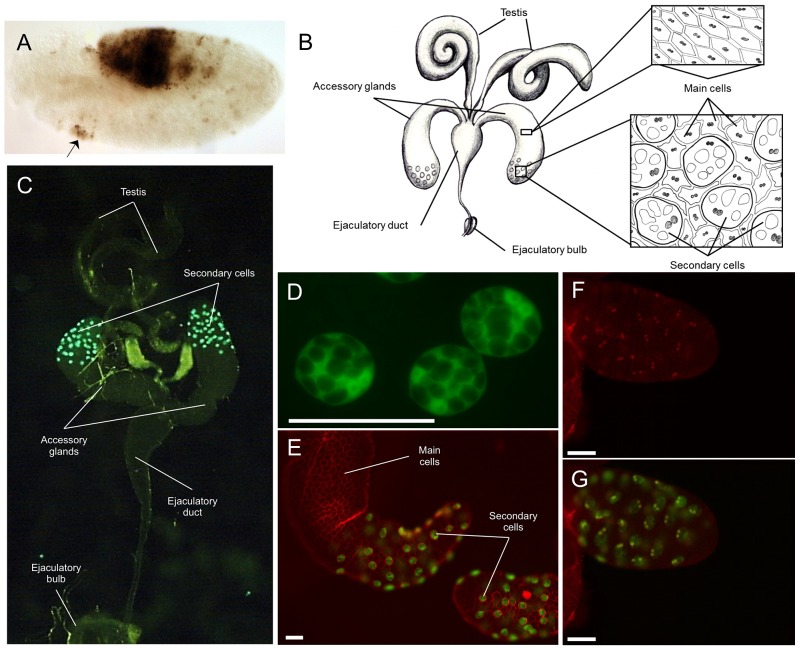
Expression patterns driven by the *Abd-B-Gal4* BAC. A) Embryo expressing the *Abd-B*-*Gal4* BAC crossed to a *UAS-LacZ* reporter stained with an antibody directed against ß-galactosidase. Out of a slight ectopic expression anteriorly (indicated by the arrow), the expression pattern is stickingly simiar to the WT *Abd-B* expression pattern as documented in Figure S2. B) Cartoon depicting the male reproductive apparatus with testis, the paired accessory glands, the ejaculatory duct and ejaculatory bulb. Each accessory gland contains two secretory cell types, the main cells which make up the majority of the gland (top insert) and the secondary cells which are located at the distal tip of the gland interspersed among the main cells (bottom insert) Drawing by J. L. Sitnik; C) Picture of the male reproductive system from flies carrying the *Abd-B*-*Gal4* BAC crossed to a *UAS-GFP* reporter with the secondary cells of the accessory glands showing GFP expression. The different organs composing the system are marked. D) Magnification of three secondary cells from flies carrying the *Abd-B*-*Gal4* BAC crossed to a cytoplasmic *UAS-GFP reporter*. The multiple, large vacuoles, characteristic of secondary cells, can be visualized through their exclusion of the GFP protein. The two nuclei of the cells can also be seen as slightly more intense GFP signals; E) The tip of the accessory gland with GFP expressed specifically in the secondary cells driven by the *Abd-B*-*Gal4* BAC (green), co-stained with the membrane staining dye, FM4–64, in red. The two cell types can be clearly distinguished with examples indicated with white lines.; F) Abd-B antibody staining of the tip of an accessory gland on *Abd-B*-*Gal4* BAC, *UAS-GFP* flies (red). Only secondary cell nuclei are stained. G) GFP expression (green) in the same gland overlaid onto the *Abd-B* antibody staining (red) shown in Figure 2F. Each cell with Abd-B protein expression also express GFP. The white scale bar on figures D, F, E, and G represents 50 µm.

To study the *Abd-B* expression pattern, a line was established containing the *Abd-B-Gal4* BAC and a UAS-GFP reporter. Initial examination of the embryonic expression pattern in these lines confirms that the *Abd-B-Gal4* BAC appears to recapitulate most of the wild-type expression pattern of *Abd-Bm* in early embryos (Figure 2A; Figure S2; Figure S3). Later, we do observe some evidence of ectopic expression from the BAC, particularly in the ventral nerve cord (Figure S2; Figure S3). Even with the slight level of ectopic expression, the *Abd-B-Gal4 BAC* seems to be a useful tool, as it recapitulates known patterns of *Abd-B* expression even in adult and larval tissues (Figure S4).

### Previously unknown location of *Abdominal B* expression in adult flies

Using this new reporter, we identified the adult male accessory gland (see Figure 2B) as a location of *Abd-B* expression (Figure 2C). More specifically, based on the expression of our *Abd-B-Gal4* BAC, *Abd-B* appears to be specifically expressed in the secondary cells (Figure 2C, 2D, and 2E). To confirm this finding, we stained accessory glands in the presence of the *Abd-B-Gal4 UAS-GFP* reporter with an antibody directed against *Abd-B* (Figure 2F, 2G). Like the reporter, accessory gland immunostaining against the *Abd-B* protein shows specific staining in the secondary cells (Figure 2F, 2G). Interestingly, we also see *Abd-B* staining in the ejaculatory duct (Figure S4C) that is not observed with our reporter (Figure 2C). This is perhaps not surprising, as the ejaculatory duct is a structure derived from the male genital disc, a tissue that primarily expresses the *Abd-Br* isoform [44] (which is also recognized by our antibody).

### Secondary cell enhancer

**Figure 3:**
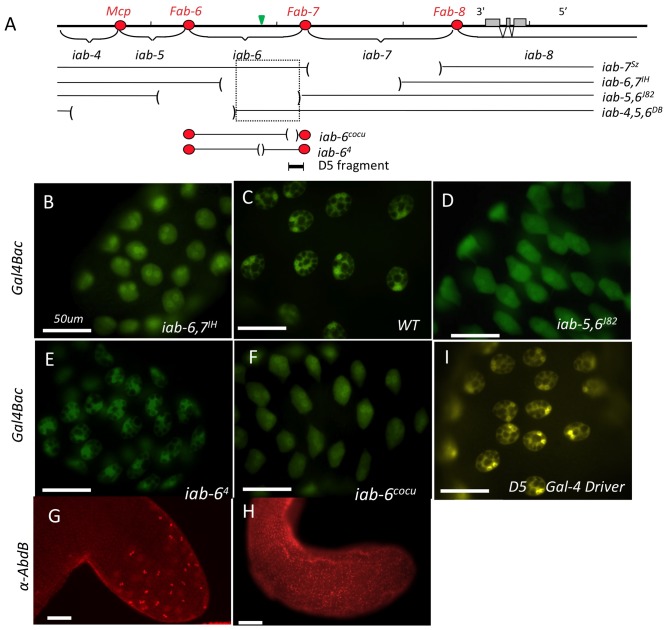
Mutants affecting *Abd-B* expression in the accessory gland. A) The Molecular map of the *Abd-B* gene region is shown with its extensive 3′ *cis*-regulatory domains *iab-5* through *iab-8* (the *iab-4* domain regulates *abd-A*). The extents of the various deficiencies that were used to map the enhancer responsible for *Abd-B* expression in the secondary cells are shown below the molecular map. The location of DNA sequence used to make the 2.8 kb-long D5rsG4rs driver (thereby refereed as D5 Gal4 driver).is shown under the map. The red circles on the map represent the boundaries separating the parasegment-specific *cis-*regulatory domains of *Abd-B*. The green triangle above the *iab-6* domain marks the *iab-6* initiator. B) UAS-GFP expression driven by *Abd-B*-Gal4 in a WT for the BX-C. C) same as B, but in an *iab-6*, *7^IH^* homozygous male or in an *iab-5,6^J82^* homozygous male(D). Note that in the *iab-6, 7^IH^* and *iab-5,6^J82^* background, the numerous vacuoles, characteristic of the secondary cells (visible by black holes in the GFP background), are lost. However, the vacuoles are not affected in *iab-4,5,6^DB^*. Thus, the critical region required for proper secondary cell specification based on these 3 deficiencies is indicated by the dotted-line box in panel A. E) UAS-GFP expression driven by *Abd-B*-Gal4 in secondary cells of *iab-6^4^* (initiator deletion) and of *iab-6^cocu^* males (F). Note the normal aspect of GFP staining in *iab-6^4^* (E) relative to the WT shown in B). In *iab-6^cocu^* however (F), the vacuoles are lost, giving rise to staining comparable to panels C and D. Panels G) and H) show *iab-6^4^* (G) and *iab-6^cocu^* (H) accessory glands stained with an *Abd-B* antibody. While *Abd-B* expression appears normal in *iab-6^4^* (G), the signal is absent in *iab-6^cocu^* (H). I) shows the tip of an accessory gland from a fly carrying the D5-Gal4 driver driving GFP expression in the secondary cells (the staining is shown in yellow to distinguish it from panels B–F depicting GFP driven by the Gal4 Bac. The white horizontal scale bars in each of the panels represents 50 µm.

In order to examine the role of *Abd-B* in the development of the accessory glands, we sought a method to remove *Abd-B* expression exclusively in the secondary cells. Rather than use the traditional FLP-FRT system for making clones, we reasoned that in our collection of *Abd-B cis*-regulatory mutations [6], we may already have a deletion that specifically removes secondary cell enhancers. Given our hypothesis that *Abd-B* might act as a cell fate determinant in the secondary cells, we screened a set of large, overlapping deficiencies covering the *Abd-B cis*-regulatory region for defects in secondary cell formation (Figure 3A). To make this analysis easier, homozygous mutant flies were screened in lines that also contain a copy of our BAC reporter to mark the cells that would normally become secondary cells. Two of the lines examined, *iab*-6,7^IH^ & *iab*-5,6^J82^(Figure 3C and 3D), showed a distinct morphological abnormality in the secondary cells. This abnormality can be easily seen using the cytoplasmic GFP marker. In wild-type cells, the GFP marker outlines the presence of large vacuolar structures in the secondary cells (Figure 2D, Figure 3B). In both the *iab*-6,7^IH^ & *iab*-5,6^J82^ mutants, these structures appear to be absent, and consequently the GFP marker is almost uniformly distributed across the cytoplasm.

Although these secondary cells are not normal, we do not detect any expression of main cell-specific markers in these cells, suggesting that they are not transformed towards a main cell fate (they still express the Acp95EF lacZ reporter gene [28] and fail to express the SP lacZ reporter gene (data not shown) [29]). To test if *Abd-B* is capable of transforming main cells into secondary cells, we expressed *Abd-B* across the whole accessory gland using a *paired-Gal4* driver [46]. The most common result of this ectopic expression is cell death in the main cells (data not shown). These results suggest that *Abd-B* expression in the secondary cells is required for morphological differentiation but may not be necessary for the initial differentiation between the two cell types.

Based on the sequences uncovered by both the *iab*-6,7^IH^ & *iab*-5,6^J82^ mutations, we concluded that the *iab-6* domain, responsible for *Abd-B* expression in segment 6, is also responsible for *Abd-B* expression in the secondary cells. Thus, we screened our collection of smaller *iab-6* deficiencies [41] for the secondary cell phenotype. From this analysis, we were able to narrow down the location containing the secondary cell enhancer to a 2.8 kb region in *iab*-6 (Figure 3A, 3F and 3H). Flies lacking this 2.8 kb region (*iab-6^Δ5^*) specifically lack Abd-B protein expression in the secondary cells (Figure 3H), and show distinct secondary cell morphological defects (Figure 3F). Like the larger deficiencies above, *iab-6^Δ5^* homozygous males lack the large vacuoles characteristic of secondary cells.

As further confirmation of the importance of *Abd-B* and the 2.8 kb *iab-6* enhancer in secondary cell development, we performed a number of control experiments. First, we crossed in a BAC transgene containing the wild-type *Abd-B* region and tested for rescue of the cellular phenotype. As expected, the secondary cells of males, homozygous for the *iab-6^Δ5^* mutation but carrying one copy of the *Abd-B* BAC are substantially rescued (containing a number of large vacuoles) (Figure S5C). Although this rescue is quite evident, it is not complete, a fact that probably reflects a weaker level of expression from the BAC relative to the native *Abd-B* locus. Indeed, *Abd-B* staining experiments using this BAC indicate that this is the case (data not shown). Next, we created a transgene carrying the 2.8 kb region of *iab-6* (called D5-Gal4) and showed that it drives expression of Gal4 in the male reproductive tract specifically in the secondary cells (Figure 3E). Using this D5-Gal4 driver, we were then able to drive expression of an Abd-B RNAi construct in the secondary cells. Knocking down Abd-B in the secondary cells was able to partially phenocopy the *iab-6^Δ5^* mutation (Figure S6C and S6F). The strength of this phenotype could be enhanced by the inclusion of a Dicer 2 overexpression transgene in the background.

*iab-6^Δ5^* was originally isolated in Iampietro et al [41], where they did not observe any visible external phenotype. With the discovery of the secondary cell phenotype and the strong reproductive phenotype described below, we have renamed this allele *iab-6^cocu^* (“cocu” means “cuckold” in French, reflecting that the mates of these males fail to reject other suitors).

### *Abd-B* expression in secondary cells is independent of the initiator

Interestingly, although the secondary cell enhancer was found in the *iab-6* domain, it does not seem to be regulated like other BX-C enhancers. Previous work has demonstrated that most enhancers in the BX-C function coordinately through their integration into segment-specifically activated chromatin domains [6], [47], [48]. A special domain control element, called an initiator, is thought to dictate the activity state of a domain along the A-P axis [41], [49], [50]. Thus, deletion of the *iab-6* initiator is predicted to inactivate *Abd-B* expression in the secondary cells, because the secondary cell enhancer should be coordinately regulated with the other enhancers in the *iab-6* domain. In contrast to this prediction, we observed that deletions of the *iab-6* initiator, which seem to show complete transformations of A6 to A5, display wild-type accessory glands (Figure 3E and 3G). From these experiments, we conclude that *Abd-B* expression in the secondary cells is set up by a different mechanism than that of tissues arising early in development.

### Impact of *iab-6^cocu^* on the production of main cell Acps

**Figure 4:**
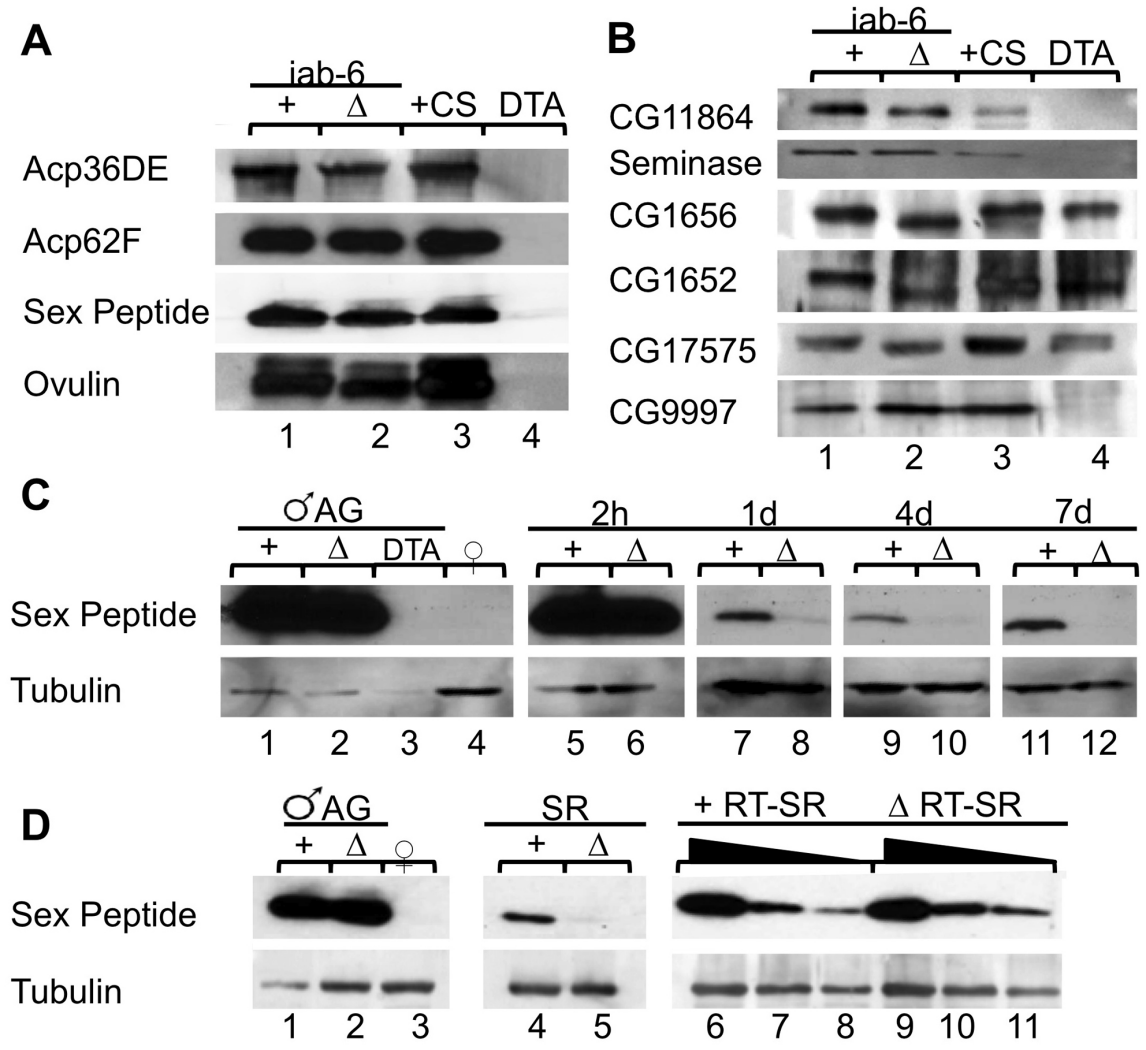
Seminal fluid proteins in *iab-6^cocu^* and control males, and their mates. A) Western blots of accessory gland extracts from two control males (lane 1), two *iab-6^cocu^* males (lane 2), two wild type males (lane 3), and two DTA-E males (lane 4). All Acps known to be produced by the primary cell (Acp36DE, Acp62F, sex peptide, and ovulin) are present in the accessory glands of *iab-6^cocu^* males, but not DTA-E males. B) Other Acps necessary for various aspects of the PMR (CG11864, Seminase, CG1656, CG1652, CG17575, and CG9997) are present in the accessory glands of *iab-6^cocu^* males. CG1656, CG1652, and CG17575 are always detectable in DTA-E males, however their abundance is highly variable compared to controls (The western blots depicted were selectived to most clearly demonstrate the presence of these proteins in DTA-E males). C) Mates of *iab-6^cocu^* males have less SP, as detected by antibodies to SP, in the reproductive tract at 1 d ASM and all subsequent time points. Tubulin was used as a loading control for the female reproductive tracts. Accessory gland extracts from a single control male (lane 1) and *iab-6^cocu^* male (lane 2) were used as a positive control and accessory gland extracts from 2 DTA-E males (lane 3) and reproductive tract extracts from 8 virgin females (lane 4) were used as a negative control. Reproductive tract extracts from females mated to either control (+) or *iab-6^cocu^* (Δ) males at 2 h (lane 5–6, 2 RTs per), 1 d (lane 7–8, 20 RTs per), 4 d (lane 9–10, 18 RTs per), and 7 d ASM (lane 11–12, 21 RTs per). D) Mates of *iab-6^cocu^* males have dramatically less SP in the seminal receptacle (SR) at 2 h ASM. Tubulin was used as a loading control for the female reproductive tracts. Accessory gland extracts from a single control male (lane 1) and *iab-6^cocu^* male (lane 2) were used as positive controls and reproductive tract extracts from 8 virgin females (lane 3) were used as a negative control. Extracts from SRs dissected from females mated to either control (+) or *iab-6^cocu^* (Δ) males at 2 h ASM (20 SRs each, lanes 4–5). The amount of SP present in the reproductive tract (minus the SR) of mates of control and *iab-6^cocu^* males was determined in a dilution series (1∶1 (lanes 6 and 9), 1∶2 (lanes 7 and 10), and 1∶4 (lanes 8 and 11) and are equivalent to 5 RTs, 2.5 RTs, and 1.25 RTs). There is no appreciable difference in the amount of transferred SP.

The *iab-6^cocu^* mutation offers the opportunity to investigate the role of the secondary cells in the PMR. First, we tested whether the main cells of these males are functional, since loss of main cell derived Acps may mask any secondary cell related phenotypes present in our mutant. We performed Western blots to examine the presence of known main cell Acps in the accessory glands of *iab-6^cocu^* males relative to two types of control males [males heterozygous for the *iab-6^cocu^* mutation (henceforth referred to as control males) and wild type males (Canton S)]. As a negative control, we included the accessory glands of DTA-E males, which lack protein production in the main cells [19] but have theoretically normal secondary cells. We used antibodies to four Acps expressed in the main cells: SP [29], Acp62F, Acp36DE [51], and ovulin; the latter Acp is also present in the secondary cells but is known to be absent in DTA-E males [52]. We detected all four Acps in the extracts from *iab-6^cocu^* males (Figure 4A). This result suggests that the main cells in *iab-6^cocu^* males are functional.

### Egg laying in mates of *iab-6^cocu^* males

**Figure 5:**
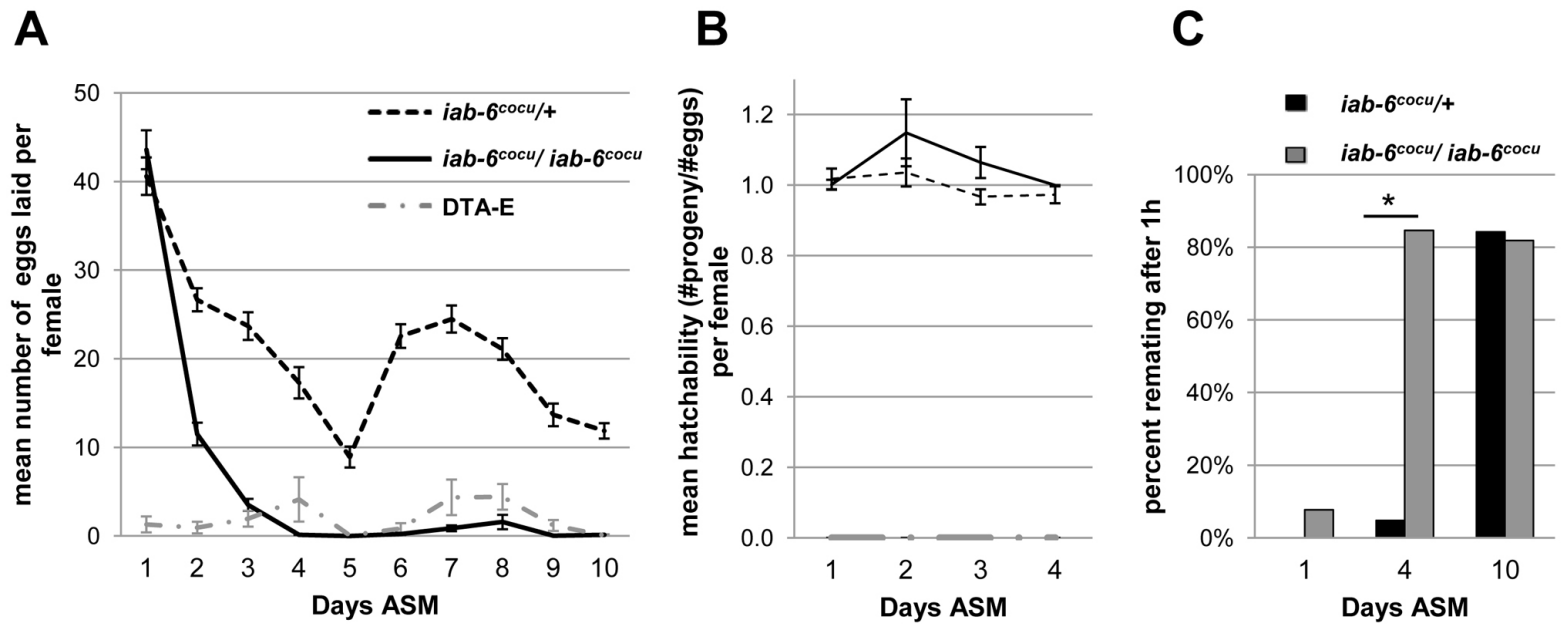
Egg-laying and receptivity in mates of *iab-6^cocu^* or control males. A) The mean number of eggs laid per female mated to either control males (dashed line), *iab-6^cocu^* males (solid line), or DTA-E males (grey dot dashed line) over a 10 day period. Mates of *iab-6^cocu^* males lay normal numbers of eggs during the first day after mating (WRST p = 0.300) but lay significantly fewer eggs over 10 days when compared to mates of control males (rmANOVA p = <0.0001*, Control N = 51, *iab-6^cocu^* N = 45, DTA-E N = 17). The drop in egg laying for controls on day 5 is atypical and was likely a response to food quality. B) The mean hatchability (#progeny/#eggs) per female for mates of control, *iab-6^cocu^*, and DTA-E males for days 1–4 of the egg laying results reported in (A). Days 5–10 were omitted because *iab-6^cocu^* mated females do not lay enough eggs on these days for analysis. Mates of *iab-6^cocu^* males have comparable hatching totals for the eggs that they do lay when compared to mates of control males (WRST p = 0.37). Because DTA-E males do not produce sperm, their mates are expected to show zero hatchability. (Values greater than 1 represent instances where the number of progeny produced exceeded the number of eggs counted; this under-counting can result when females lay eggs under bubbles in the medium or directly on top of previously laid eggs. Hatchability values were not normalized to 1 so as to accurately report counter error.) C) The percentage of mated females willing to mate within 1 hour of exposure to a wild type male at 1, 4, and 10 days after an initial mating. Both groups of females initially mated to *iab-6^cocu^* or control males are unreceptive (WRST p = 0.21, control n = 20, *iab-6^cocu^* n = 26). At 4 d ASM females initially mated to *iab-6^cocu^* males are significantly more receptive to courting males compared to mates of control males (WRST p = <0.0001*, control n = 21, *iab-6^cocu^* n = 26). By 10 d ASM there is no difference between mates of control or *iab-6^cocu^* males (WRST p = 0.84, control n = 19, *iab-6^cocu^* n = 22).

To test if the *iab-6^cocu^* mutation impacts the ability of males to induce egg-laying in their mates, we crossed *iab-6^cocu^* males, control males, and DTA-E males to virgin females. During the first 24 hours after the start of mating (ASM), the number of eggs laid by females that had mated to *iab-6^cocu^* males is comparable to that of mates of control males and is significantly higher than the number laid by females mated to DTA-E males (Figure 5A). This indicates that the *iab-6^cocu^* mutation does not impact the STR and supports the Western blot results that suggest that the main cells are normal. However, egg laying in mates of *iab-6^cocu^* males decreased dramatically at 48 hours, and the total number of eggs produced over the entire 10 day period was significantly lower than the number laid by mates of control males (Figure 5A) (note that Canton-S males behave similarly to our control males (Figure S7)). This drop in egg laying is consistent with that observed when females do not receive or fail to store/release SP. This suggests that products from the secondary cells may be necessary for maintenance of the LTR. The proportion of progeny that eclosed from eggs laid by females mated to either *iab-6^cocu^* or control males was comparable, suggesting that there is no effect of secondary cell products on hatchability (Figure 5B). Together these results suggest that the secondary cells perform a function that is essential for the maintenance of the post-mating egg-laying increase, but does not impact hatchability, and that this function is perturbed in *iab-6^cocu^* males.

### Receptivity in mates of *iab-6^cocu^* males

Under normal conditions, mated females are less receptive to subsequent mating for more than four days after the initial mating occurred [53]. This reduction in receptivity requires the receipt of Acps [19], [23], [26]. To test whether the *iab-6^cocu^* mutation alters female receptivity to remating, we mated virgin females to either *iab-6^cocu^* or control males and then allowed these females access to a single WT male at 1 d, 4 d, or 10 d ASM. At 24 hours after the initial mating, neither group of females remated, further suggesting that the STR is intact in mates of *iab-6^cocu^* males. However, when mated females were introduced to a WT male at 4 days ASM, females which had initially mated to *iab-6^cocu^* males were significantly more receptive than mates of control males. At 10 days ASM, both groups were fully receptive (Figure 5C). Our results show that sexual receptivity is initially repressed in mates of *iab-6^cocu^* males, but that this effect is not maintained. This finding demonstrates a defect similar to those observed in known LTR-related proteins and further corroborates the LTR phenotype observed in our egg-laying experiments.

To verify that both the egg-laying and receptivity phenotypes are caused specifically by the loss of Abd-B expression in the secondary cells, we also performed these experiments using BAC rescued *iab-6^cocu^* flies (Figure S5) and D5-Gal4::*Abd-B* RNAi flies (Figure S6). In both cases, these experiments confirm a role for *Abd-B* in producing the PMR phenotypes. Again, as with the cellular phenotypes mentioned above, both the rescue and phenocopying was incomplete, though clearly significant. Given the caveats involved in these experiments regarding the level and timing of protein expression, this was perhaps not unexpected and demonstrate a strong relationship between the celluar and the behavioral phenotypes. Nonetheless, these data clearly point to a major role for *Abd-B* expression in the secondary cells in modulating the PMR.

### The production of LTR-associated Acps in *iab-6^cocu^* males

Our results suggest that the secondary cells are necessary for the processes required for long-term PMR maintenance. Therefore, the *iab-6^cocu^* mutation likely impacts proteins required for the LTR. While the Acps that are produced and transferred to females have been extensively described, [51], [54]–[57] little is known about their cellular origins. Thus, we investigated the possibility that some of the known PMR-related Acps, and more specifically those involved in the LTR, could be produced in the secondary cells or in both cell types, and thus, may be absent in *iab-6^cocu^* males.

We performed Western blots to examine the presence of known LTR Acps in the accessory glands of *iab-6^cocu^* males relative to control males. As a negative control for main cell expressed Acps, we included DTA-E males. Any secondary cell-expressed Acp should still be produced in these males, but main cell expressed Acps should not. We used antibodies to six Acps that regulate the PMR; one STR associated Acp (CG11864) and five LTR associated Acps (CG10586 (Seminase), CG9997, CG17575, CG1656, and CG1652 [36], [38]–[40]). All of these Acps were present in *iab-6^cocu^* males (Figure 4B). Surprisingly, three of the Acps associated with the SP pathway (CG17575, CG1656, and CG1652) were also present in AG extracts from DTA-E males suggesting that they may be secondary cell expressed. Supporting this hypothesis, RNAi of these proteins in the secondary cells knocksdown their expression, while leaving a main cell-expressed control protein, Acp62F, unchanged (Figure S8). The remainder of the Acps, CG9997, CG11864, and Seminase, are likely all expressed primarily or exclusively in the main cells. Further, these results support our previous conclusion that the secondary cells in *iab-6^cocu^* males maintain a distinct expression profile from main cells and still produce some secondary cell proteins.

### A role for secondary cells in SP storage

The LTR defects seen in mates of *iab-6^cocu^* males are consistent with those associated with failure to store or release SP [51], [55]–[57]. However, *iab-6^cocu^* males produce SP and all known LTR Acps. Still, it is possible that the *iab-6^cocu^* mutation interferes with the ability of SP to enter storage and thus maintain the LTR. We tested for this by performing Western blots using SP antibodies. Both control and *iab-6^cocu^* males transfer SP to their mates, and there are comparable amounts of SP in the female reproductive tract by 2 h ASM. However, by 24 hours ASM and continuing to 6 days thereafter, mates of *iab-6^cocu^* males have significantly less stored SP (Figure 4C; Figure S9). To distinguish between premature loss of SP from the seminal receptacle (SR) versus failure of SP to be stored in the SR initially, we performed Western blots of SRs dissected from females mated to either *iab-6^cocu^* or control males. Significantly less SP is present in the SR of mates of *iab-6^cocu^* males at 2 h ASM compared to mates of control males, while the amount of SP present in the remainder of the reproductive tract is comparable, though slightly higher in mates of *iab-6^cocu^* males (Figure 4D; Figure S9). These results suggest that *iab-6^cocu^* males transfer normal amounts of SP but that this SP fails to enter the SR. The reduction/absence of stored SP at later time points (1–7 days ASM) is likely responsible for the reduction in egg laying and the increase in receptivity seen in mates of *iab-6^cocu^* males.

### Sperm competition in mates of *iab-6^cocu^* males

**Figure 6:**
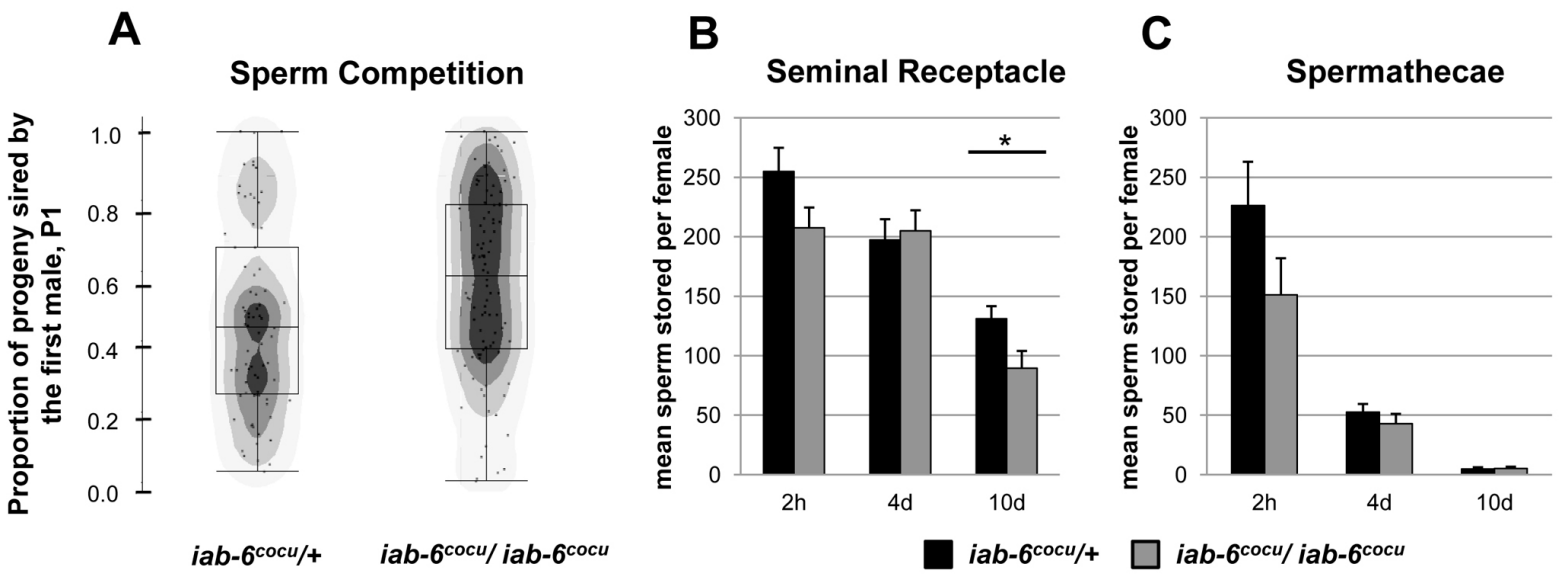
Sperm storage and use by mates of *iab-6^cocu^* or control males. A) For sperm competition assays *cn bw* females were first mated to either control (left) or *iab-6^cocu^* (right) as the first male and allowed to mate a second time with a *cn bw* male. The proportion of progeny sired by *iab-6^cocu^* males when acting as the first male (P1, # progeny from first male/total progeny) was significantly higher when compared to females who first mated with control males (WRST p = 0.038*, control N = 74, *iab-6^cocu^* N = 98). B&C) Counts of sperm stored in mates of control (black) and *iab-6^cocu^* (grey) males at 2 h, 4 d, and 10 d ASM. B) Mates of *iab-6^cocu^* males have wild type numbers of sperm present in the seminal receptacle at 2 h (WRST p = 0.10, control N = 8, *iab-6^cocu^* N = 11) and 4 d ASM (WRST p = 0.96, control N = 10, *iab-6^cocu^* N = 8) but fewer at 10 d ASM (WRST p = 0.017*, control N = 19, *iab-6^cocu^* N = 12) when compared to mates of control males. C) Mates of *iab-6^cocu^* males show wild type numbers of sperm stored in the spermathecae at all time points. 2 h (WRST p = 0.13, control N = 7, *iab-6^cocu^* N = 10); 4 d (WRST p = 0.38, control N = 10, *iab-6^cocu^* N = 7); 10 d (WRST p = 0.77, control N = 17, *iab-6^cocu^* N = 16).

SP also plays a role in sperm competition [58], which occurs when ejaculates from two males are present within the same female reproductive tract [59]. For example, in circumstances where SP null males are the first male to mate with a given female, they sire a higher percentage of the total progeny (P1, #progeny from first male/total progeny) than control males [33]. To test whether the *iab-6^cocu^* mutation also affects P1, we mated *iab-6^cocu^* and control males to *cn bw* females and, after 3 days, allowed them to mate with *cn bw* males. The *iab-6^cocu^* males had significantly higher P1 than control males consistent with a problem in SP presence or storage (Figure 6A).

### Sperm storage in mates of *iab-6^cocu^* males

One possible explanation for the reduction in stored SP in mates of *iab-6^cocu^* males is a defect in sperm entry into storage or an increase in the rate at which sperm are released from storage. To test this, we counted sperm present in both female sperm storage organs at 2 hours, 4 days, and 10 days ASM. Females mated to either control or *iab-6^cocu^* males store sperm at comparable levels at 2 hours ASM and appear to retain normal numbers through 4 days ASM (Figure 6B and 6C). These results suggest that initial sperm storage and release is normal in mates of *iab-6^cocu^* males and that the reduced level of SP in the seminal receptacle at 2 hours ASM is not due to a failure to adequately store sperm. Mates of *iab-6^cocu^* males do not show the stereotypical sperm over-retention phenotype seen with knockdown of other LTR related proteins [33] and instead show a slight but significant decrease in stored sperm within the SR at 10 days ASM when compared to controls (Figure 6B). This is not wholly surprising, as the *iab-6^cocu^* mutation does not result in the absence of a single gene product, but likely several that contribute to different aspects of the PMR. It is possible that the secondary cells produce, modify, or transfer some product necessary for sperm to be retained within the female sperm storage organs. When SP is absent, this product may be regulated improperly resulting in the typical sperm over-retention phenotype. A loss of, or reduction in this product, combined with the SP retention defect, may explain these results.

### Several Acps display defects in glycosylation, stability, or protein abundance within the reproductive tract of females mated to *iab-6^cocu^* males

**Figure 7:**
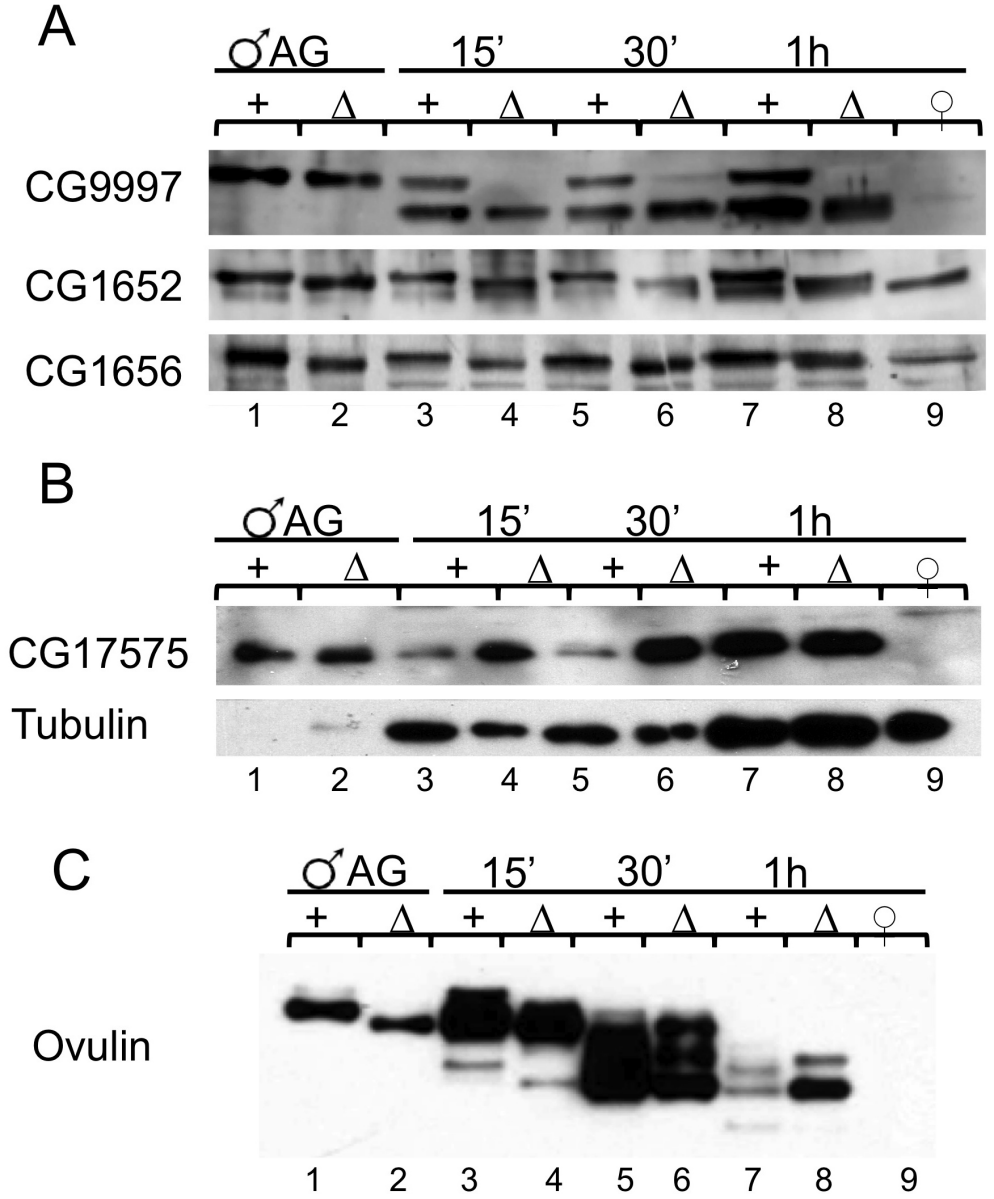
Post translational modification, stability, and abundance of seminal fluid proteins in mates of *iab-6^cocu^* or control males. Western blots using antibodies of known LTR-associated Acps CG9997, CG1656, CG1652, and CG17575 as well as STR Acp ovulin (Acp26Aa). Accessory gland extracts from a single control (lane 1) and *iab-6^cocu^* male (lane 2) were used as positive controls and reproductive tract extracts from 4 virgin females (lane 9) were used as a negative control. Extracts from the reproductive tracts of females mated to control (+) or *iab-6^cocu^* (Δ) were collected at 15′ (lanes 3–4, 2 RTs per), 30′ (lane 5–6 s, 3 RTs per), and 1 h ASM (lanes 7–8, 6 RTs per). A) Full length CG9997 is produced by *iab-6^cocu^* males but is not present in the reproductive tracts of their mates. The smaller processed form of CG9997 is present in mates of *iab-6^cocu^* suggesting that CG9997 is transferred. Both CG1656 and CG1652 are transferred to females normally by *iab-6^cocu^* males, but both of these proteins run at a lower apparent molecular weight than in control males. B) *iab-6^cocu^* males transfer more CG17575 to their mates than control males. Tubulin was used as a loading control for the female reproductive tracts. C) Both mates of control and *iab-6^cocu^* males receive ovulin. However, the ovulin produced by *iab-6^cocu^* males also runs at a lower apparent molecular weight than in controls.

Our Western blots and RNAi data showed that three of the known LTR specific proteins (CG1656, CG1652, and CG17575) are produced in the secondary cells and that one (CG9997) is likely produced by main cells (Figure 4B and Figure S7). We considered the possibility that failure to transfer one or all of these Acps to the female during mating could contribute to the SP storage defect seen in mates of *iab-6^cocu^* males. To test this, we performed Western blots on the reproductive tracts of females mated to either *iab-6^cocu^* or control males at 15 m, 30 m, and 1 h ASM using antibodies to CG9997, CG1656, CG1652, and CG17575. Although all four Acps are transferred to females and are present in the female reproductive tract at all time points tested, their abundance, gel mobility, or processing appear to be abnormal in mates of *iab-6^cocu^* males (Figure 7).

### *iab-6^cocu^* males carry out abnormal glycosylation of several Acps

Both CG1656 and CG1652 run at a lower apparent molecular weight in *iab-6^cocu^* males compared to control males (Figure 7A, Figure 4B). Ovulin, likewise, shows reduced apparent molecular weight in *iab-6^cocu^* males (Figure 7C, Figure 4A). The gel mobility differences for these proteins in *iab-6^cocu^* versus control males is evident both within AG extracts as well as across time points (15 m, 30 m, and 1 h ASM) within the female reproductive tract of their mates. Ovulin is normally processed inside the female reproductive tract [60]. This processing appears to occur properly in mates of *iab-6^cocu^* males, although the apparent molecular weight of some cleavage products is altered. It is unlikely that these differences in apparent molecular weight are caused by sequence differences or background effects. The controls used for these experiments are heterozygous for all of the chromosomes in the *iab-6^cocu^* mutant line. Thus, if the gel mobility differences were caused by sequence differences, we would expect to see two bands indicating the WT and altered version of each protein. Further, ovulin and CG1656/1652 are located on separate arms of chromosome 2 and are necessary for different aspects of the PMR. Together, these observations suggest that the gel mobility differences may be a result of posttranslational modification.

**Figure 8:**
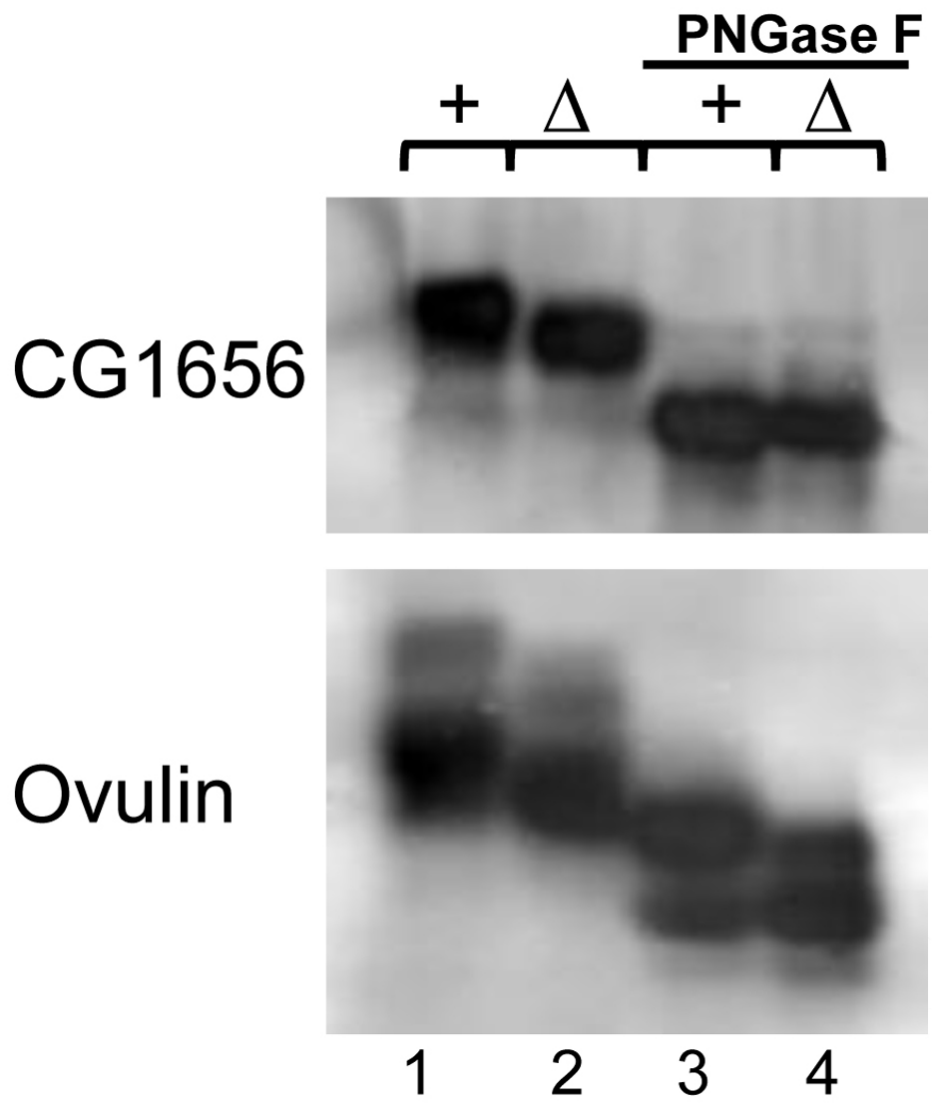
Glycosylation measurements of seminal fluid proteins in *iab-6^cocu^* or control males. PNGase F assays were used to examine glycosylation states of CG1656 and ovulin or determine whether N-linked glycosylation differences underlie the gel differences seen between control (+) and *iab-6^cocu^* (Δ) males. Untreated (lanes 1–2) and PNGase F treated (lanes 3–4). In both cases the gel mobility differences seen between control and *iab-6^cocu^* males are absent after PNGase F treatment suggesting that in *iab-6^cocu^* males both proteins are improperly glycosylated.

Ovulin is a glycoprotein [52], but little is known about the posttranslational modifications of CG1656 and CG1652. To test whether differences in glycosylation underlie the gel mobility differences observed, we treated extracts from control and *iab-6^cocu^* males with PNGaseF. This treatment, which removes N-linked glycosylation [61], [62], resulted in loss of the apparent molecular weight differences between *iab-6^cocu^* and control flies for all three proteins (Figure 8, CG1652 not shown). These results suggest that the secondary cells contribute to the regulation of posttranslational modifications, and more specifically glycosylation, of Acps. They are also the first evidence of the presence of N-linked glycosylation on CG1656 and CG1652.

To verify that the glysocylation differences are caused specifically by the loss of Abd-B expression in the secondary cells, we also looked at the gel mobility of CG1656 and CG1652 in BAC rescued *iab-6^cocu^* flies (Figure S5, CG1652 not shown). The rescue BAC was able to restore proper glycosylation in the AGs of some males but not others. This is consistent with the partial rescues we have observed, especially if the glycosylation phenotype is dose dependant and the BAC males are on the threshold. Still, these results support the connection between Abd-B expression in the secondary cells and proper glycosylation of Acps and suggests that there may be a connection between these glycosylation differences and the PMR.

### Mates of *iab-6^cocu^* males display abnormal stability or abundance of some Acps

In wild type males, CG9997 is transferred to females as a full length protein (45 kD) and is processed in the female reproductive tract to a smaller form (36 kD). Both products persist in the female for longer than 1 h. Males with the *iab-6^cocu^* mutation produce full length CG9997 but the full length form does not persist inside the female reproductive tract (Figure 7A). A similar increase in processing or instability of the full length product was seen in males that do not produce or transfer CG1656/CG1652 [39]; its biological relevance is currently unknown. Since *iab-6^cocu^* males produce and transfer CG1656/CG1652, our results suggest that in addition to these two proteins, the products of the secondary cells are essential for regulating the stability of CG9997 inside the female. As observed with the other LTR Acps we assayed, CG17575 is produced and transferred to females by *iab-6^cocu^* males. However, CG17575 is present in higher amounts in the reproductive tract of females mated to *iab-6^cocu^* males at all time points when compared to controls (Figure 7B; Figure S9). Whether this difference is due to increased transfer or failure to degrade CG17575 within the tract is unclear.

## Discussion

Here, we report that *Abd-B* is expressed in the secondary cells of the *Drosophila* male AG and, using mutations that specifically remove *Abd-B* from these cells, uncover roles for this previously unstudied but important reproductive cell type. Furthermore, we show that *Abd-B* expression in these mesodermally-derived cells does not fit the “initiator” paradigm developed for the segment-identity function of Hox genes in ectodermal tissues. And finally, we demonstrate that the secondary cells of the male AG synthesize products necessary for maintenance of the seminal fluid's effects on the female PMR.

### New insights into *Abd-*B gene regulation

Due to the large size and complexity of the *Abd-B* regulatory region, we created a BAC- reporter construct to monitor *Abd-B* expression in the adult fly. When combined with fluorescent markers, this method allowed us to bypass the technical issues of antibody penetration and the laborious dissections needed for *in situ* hybridization or immunohistochemistry to identify a novel area of *Abd-B* expression in the adult. Overall, the BAC reporter is able to accurately reproduce the known, complex *Abd-B* expression pattern; indeed, our BAC construct seems to more-faithfully reproduce *Abd-Bm* expression than even a previously isolated transposon insert in the *Abd-B* promoter (*Abd-B-Gal4^LDN^*) [63]. Furthermore, by combining our BAC reporter with pre-existing deletion mutations, we were able to discover the function of a vital gene in an adult tissue without the need to create mitotic clones.

From the standpoint of Hox gene regulation, our discovery of the secondary cell enhancer is quite interesting because, unlike other cell-type specific enhancers from the BX-C, the secondary cell enhancer does not seem to be regulated by a domain initiator [6], [41], [49]. Most cell-type specific enhancers from the BX-C are not intrinsically restricted along the A-P axis. They are restricted only to a specific cell-type and gain A-P restriction through clustering in a BX-C domain. For example, in a transgene assay, an *Abd-B* enhancer from the *iab-7* domain (called 11X) drives expression in the tracheal placodes in all segments. However, in the BX-C, this enhancer seems to be active only in the *Abd-B* expression domain [6]. The clustering of enhancers to one area of the chromosome is thought to allow all of the enhancers to be coordinately regulated along the A-P axis as a domain through the changing of the local chromatin environment. The *Polycomb (Pc)* repression machinery is thought to be critical for this process by creating repressive chromatin over inactive domains ([6] and refs. therein). Specialized elements, called initiators, seem to read an A-P segmental address and act as domain activators, probably by preventing *Pc* repressive complexes from establishing on active domains [41].

The domain model predicts that deletion of an initiator element should prevent domain activation, leaving all enhancers in its domain inactive [41]. Based on this paradigm and the fact that the secondary cell enhancer was found in the *iab-6* domain, we expected that the deletion of the *iab-6* initiator would abolish *Abd-B* expression in the secondary cells. However, we found that *Abd-B* expression in the secondary cells of initiator mutants was normal. This finding argues against the strictest interpretation of the initiator model. We can propose several hypotheses to resolve this discrepancy. For example, the *Pc* repression system is known to act on many genes during development, but its main targets appear to be the homeotic genes during the establishment of segment identity. It is possible that late in development, after cells have made initial cell fate decisions (and the segment identity role of the homeotic genes might be less important), the targets of *Pc* silencing might change. Such loosening of *Pc* silencing over the *Abd-B cis*-regulatory regions would allow previously silenced enhancers to become available for regulating *Abd-B* expression so that it could perform other functions, such as in the secondary cells.

Alternatively, the difference in *Abd-B* gene regulation that we observe in secondary cells may reflect the cellular origin of the secondary cells. Most BX-C *cis*-regulatory mutations were isolated based on cuticular phenotypes and confirmed using antibody staining in the epidermis and CNS. These tissues are of ectodermal origin, unlike the mesodermally-derived secondary cells [64]. Perhaps, the rules governing the coordination of Hox expression in the ectoderm do not hold true in the other germ layers. Consistent with this, BX-C genes are expressed differently in the gut visceral mesoderm than in the ectoderm [65].

Evolutionary considerations may provide some explanation for why the fly uses different means of controlling *Abd-B* expression in embryonic segment identity specification *vs.* in later reproductive tissues. *Abd-B* class Hox proteins play roles in the formation of the external genitalia in both arthropods and mammals [66]–[70]. Due to the similarity in expression pattern and function, it has been proposed that *Abd-B*'s role in the formation of the genitalia predates its role in segmental identity [67] [71]. Here, we have shown that *Abd-B* is also important for correct development of cells within the *Drosophila* male AG that produces many seminal fluid proteins required for male reproductive success. The mammalian orthologues of *Abd-B*, the Hox9 to 13 class of genes, are expressed in the developing seminal vesicle and prostate gland, both seminal protein secreting organs [72], [73]. The analogy in function between these organs, and their similarity in gene expression patterns suggests that the role of *Abd-B* class genes in the male reproductive tract might be an ancient, conserved function, potentially independent of its role in segmental identity. In this light, it would not be surprising that *Abd-B* regulation in the secondary cells escapes the domain regulation seen for *Abd-B* function in segment identity determination. The *cis*-regulatory domains for segment identity could have been added separately, possibly through co-opting the *abd-A* gene regulatory regions, as suggested by transvection studies [74], [75]. In any case, the adding of *cis*-regulatory sequences and the consequent constraints of the domain model on *Abd-B* would necessarily have to preserve its late function in the secondary cells.

### The function of the secondary cells of the male accessory gland

Previous studies have shown that the male AG produces Acps required to initiate and maintain a range of PMRs in females. Further, diptheria-toxin mediated-ablation of accessory gland main cells demonstrated that products of these cells are essential for the PMR. The importance of the main cells was further strengthened by the discovery that they produce the Acp sex peptide (SP), which is essential for many aspects of the PMR and whose persistence allows maintenance of PMR effects (i.e. the LTR) [29]. Additional Acps important for other aspects of the PMR (e.g. Acp36DE, ovulin) were also found to be produced by main cells. Thus, until now, the role of the secondary cells was unknown and no methods to directly target these cells were available.

**Figure 9:**
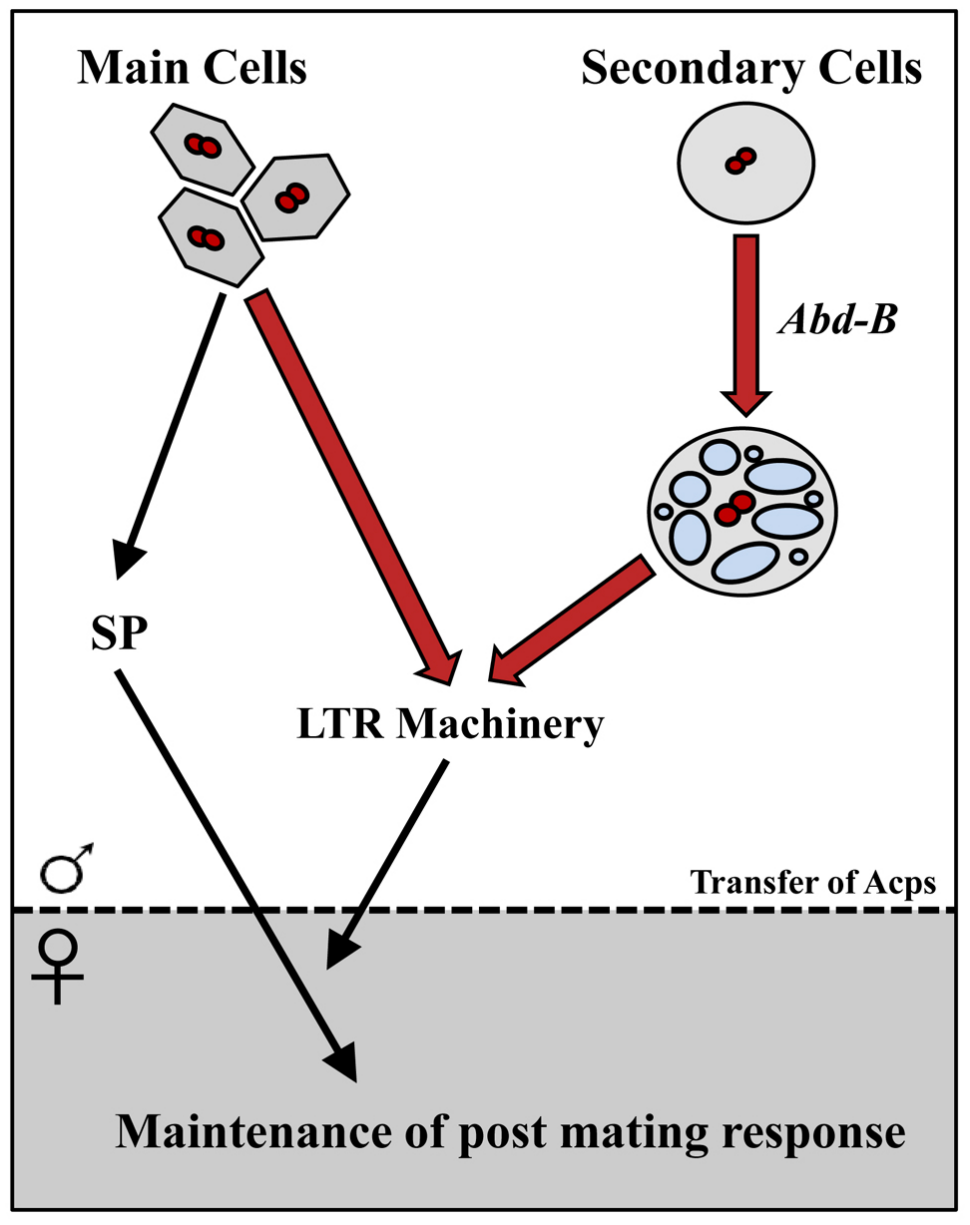
Summary/model. Bold arrows delineate the new findings in this paper. The Drosophila male accessory gland consists of two secretory cell types: main cells and secondary cells. Main cells produce seminal proteins essential for inducing post-mating responses (PMR) in mated females; the function (if any) of secondary cells was unknown. Sex Peptide (SP) from the main cells induces many aspects of PMR, but persistence of its effects requires other seminal proteins (the LTR machinery: CG9997, CG17575, CG1652 and CG1656), whose cellular source was unknown. Here, we showed that the Hox gene *Abd-B* is essential for normal development of the secondary cells, but with regulatory characteristics (not shown in this figure) that differ from those used in segment identity. By deleting the secondary-cell regulatory element of *Abd-B*, we obtained males with abnormal secondary cells. Mates of those males failed to maintain the PMR, indicating that secondary cells play an essential role in reproduction: their products, along with main cell products, allow persistence of SP in mated females, thus prolonging their post-mating responses.

Using the *iab-6^cocu^* regulatory mutant of *Abd-B*, we demonstrated that the secondary cells make a unique contribution to maintenance of the female's post-mating changes in egg-laying, receptivity, sperm competition, and sperm storage (summarized in Figure 9). Our results are consistent with findings about secondary cell function obtained independently by Minami et al., from their study of *dve* mutants [76]. The inability of *iab-6^cocu^* males to maintain the PMR in their mates is consistent with perturbation of the function of the “LTR network”. These LTR network proteins are needed to promote the association of SP with sperm in the SR, an association that is required for SP-mediated maintenance of the PMR [22], [39]. The results of our study also show that three of these LTR network Acps, CG1656, CG1652, and CG17575 are all produced in the secondary cells while CG9997 and Seminase are primarily or exclusively main cell expressed. This is the first direct evidence that Acps from both cell types work together in a complex pathway. While all reported LTR specific network proteins (CG9997, CG1656, CG1652, and CG17575) are present in the *iab-6^cocu^* mutant, they are all abnormal, either in amount/stability inside the female reproductive tract or in glycosylation state; how these changes result in the SP storage defect is an important area for future study.

The phenotype of *iab-6^cocu^* males shows one exception to the standard LTR phenotype. Mates of SP null males (or males knocked down for any of the other 4 LTR related proteins) have high rates of sperm retention; this phenotype is lacking in mates of *iab-6^cocu^* mutants. The mechanisms behind the release of sperm from storage and this sperm retention phenotype are unknown. Loss of *Abd-B* expression most likely impacts a wide variety of Acps and potentially cellular functions associated with vacuoles (such as transmembrane transport). Thus, it is possible that one or a combination of the proteins affected by this mutation (some as yet unidentified) may negatively impact normal sperm storage independent of the influence of *Abd-B* on the storage of SP in the seminal receptacle. This could result in masking the over-retention of sperm seen in SP nulls due to the loss or abnormal function of a protein or proteins necessary for the retention phenotype to occur. Further work investigating the role of individual secondary cell associated Acps in the LTR, as well as sperm storage, may be helpful in determining how these processes function.

The *iab-6^cocu^* mutant revealed another unique role for secondary cells: regulating glycosylation of at least three seminal proteins (ovulin, CG1656, and CG1652); the impact of glycosylation on the functions of these proteins is as yet unknown. Our findings show that CG1656 and CG1652 are produced by the secondary cells. However, ovulin is produced in main cells as well as secondary cells, and is detected in the AG lumen as well as in the vacuoles of the secondary cells [52]. As such, we suggest three possibilities for how secondary cells might mediate glycosylation: 1) through the secretion of glycosylation substrates that can be taken up and used by main cells, 2) through the secretion of glycosyation regulators directly into the lumen where they could modify Acps from both cell types that are present there, or 3) that Acps like ovulin and other main cell products are taken up into these vacuoles and then modified before being secreted into the lumen as mature, glycosylated proteins. Future dissection of the glycosylation phenotypes in the *iab-6^cocu^* mutant males will help shed light on the role glycosylation plays in regulating the PMR and how this process is regulated in the tissue as a whole.

It is important to note that our *Abd-B* regulatory mutant still has secondary cells, or at least precursor cells poised to become secondary cells. The initial differentiation step that allows for *Abd-B* expression in the secondary cells is not perturbed by the *iab-6^cocu^* deletion. As such, although we have already found a new and unanticipated role for secondary cells in regulating the PMR, it is possible that these cells play additional roles. Future cell ablation experiments using tools derived from this study will allow tests of such additional roles.

In conclusion, we have shown that each of the two cell types of the *Drosophila* AG plays important roles in producing the female PMR: the main cells producing several Acps to initiate and maintain the PMR, and the secondary cells providing products to aid in temporally extending the response. We expect that through the action of various combinations of transcription factors, like *Abd-B*, the two cell-type lineages have diverged into distinct, and specialized cell-types. Although these two cell types perform vitally intertwined functions, they are maintained as separate cell types. This suggests that there may be a requirement for compartmentalization of their functions or products, or that the two cell types evolved separately for some other purpose and were functionally associated afterward. It is, therefore, of great future interest to identify the specific products of each of these cell types and to determine how they work in conjunction to mediate the full reproductive effect of seminal secretions.

## Materials and Methods

### Creation of *Abd-B* rescue BAC

The *Abd-B* rescue BAC was constructed from BACR24L18 (size - 171936 bp; AC095018), which contains the *Abd-B* region of the BX-C. We first constructed a pW25-based vector [77] to be used to add sequences to the BAC for site-specific integration into the *Drosophila* genome. The original *white* gene carried by pW25 was replaced by a *Su(Hw)* insulator-flanked *white* gene from the SUPor-P plasmid [78] by amplifying it with *NotI-forward* and *AscI-PmeI reverse* (see Table 1 for primer list). These primers carry restriction sites for insertion of the product into pw25 after appropriate enzyme digestion. A fragment containing the kanamycin resistance gene (KanR) was amplified from pIGCG21 [79] using the following primers: PmeI5′Kan, 5′attB3′KanAS. Next, an AttB sequence was PCR amplified using the primers 3′Kan5′attBS, and PmeI3′attBAS. The KanR and AttB fragments were then mixed together with the PmeI5′Kan and PmeI3′attBAS primers for a final overlap PCR reaction. The resulting PCR fragment, containing the KanR gene and an AttB sequence flanked by PmeI sites, was cloned into pGemTeasy. After sequencing, this fragment was excised with PmeI and cloned into the unique PmeI site of the modified pW25 vector (resulting in pW25/Kan-AttB).

**Table 1:** List of primers used to perform the different constructs described in Materials and Methods.

Name	Sequence
NotI-forward	AAAAAGCGGCCGCGCGATAATCATTTAATAGTCGAC
AscI-PmeI reverse	AAAAAGGCGCGCCGTTTAAACCGAAATGCGTCGTTTAGAGCAG
PmeI5′Kan	GTTTAAACTGCAAGGCGATTAAGTTGGG
5′attB3′KanAS	CCGTGACCTACATCGTCGACGGTACCTGCTCAGAAGAACTCGTCAAGAAGGCG
3′Kan5′attBS	CGCCTTCTTGACGAGTTCTTCTGAGCAGGTACCGTCGACGATGTAGGTCACGG
PmeI3′attBAS	GTTAAACGGCTTGTCGACATGCCCGCCG
NotI iab4	AAAAGCGGCCGCGGCGCTAAATGAATCCACCCACG
EagI iab4	AAAACGGCCGGCCACATGGATGCCTCGTCAAG
MluI SacBII	AAAACGCGTGCCAACACGGGAACAGAAAACGG
AscI SacBII	AAAAGGCGCGCCCCTCGAAGAAGCCTCTGTTTGTC
Amp BamHI	AAAAAGGATCCCTCATGAGACAATAACCCTG
Amp XmaI	AAAAACCCGGGCTTGGTCTGACAGTTACC
NHR L frw NotI/long	AAAAAGCGGCCGCAAGCGGCCCTGCAACTTCGTCGAGGACTGGGACTTGAACG
NHR L rev OL/long	TGCATTCTAGATTTTTGAATTCGACGGGCAGGGAGGGATGCGCCTGGGGATGCGGATG
NHR R frw OL/long	CCGTCGAATTCAAAAATCTAGAATGCAGCAGCACCATCTGCAGCAGCAGCAACAGCAGCAGCAGCAG
NHR R rev XmaI/long	AAAAACCCGGGTTGTTTGTCGATGTCATGGATGTGGGTGCATTCACACCTC
NSx/A new F EcoRI	AAAAAGAATTCCAGCTCAACAGTCACACATAGACAG
NCSx/A new R XbaI	AAAAATCTAGAACGAGTTCTTCTGAGCGGGACTCTG
F300 NS/A rec	GTCACTCAGAGGAGTGAGAA
R300 NS/A rec	TGTACGGCGACAAGTGGCAC
HR-R Gal4rep	ACCCACCGCCCCGCACCCGCATCCGCATCCCCAGGCGCATCCCTCCCTGCCCGTCATGAAGCTACTGTCTTCTAT
HR-L Gal4rep	GCTGCTGCTCCTGCTGCTGCTGCTGCTGTTGCTGCTGCTGCAGATGGTGCTGCTGTTCGCTATTACGCCAGCCCG
D5 F	AAAAAGGATCCCAGGAGCAATCCATCAAA
D5 R	AAAAAGGATCCACAGCTCTGCTTTTTGCTGA

To mediate recombination in bacteria, two homology regions were then added to this vector. First, an 859 bp fragment from the *iab4* region (*iab4* HR: 101409–102267 coordinates in the BACR24L18) was made by PCR with the primers: NotI *iab4* and EagI *iab4*. The resulting PCR fragment was cut with NotI and inserted into the unique NotI site of the modified pW25. A second homology fragment was designed to target the SacBII gene present on the BAC backbone. Using the PCR primers: MluI SacBII and AscI SacBII, a 525 bp fragment from SacBII was amplified (SacBII HR: 921–1445 coordinates in the BACR24L18). The resulting 525 bp fragment was digested with AscI and MluI, and inserted into the unique AscI site of pW25/Kan-AttB. Clones for both homology regions were selected in an orientation required by the homologous recombination process to function correctly. The completed construct was digested with the ISce-I endonuclease and the fragment containing the homology regions flanking the KanR gene, the white gene and the AttB site was gel purified. This fragment was then used to recombineer BAC24L18 using the protocol of Soren Warming [80]. The resulting BAC, called *iab4-SacBII BACR24L18D* (108528 bp), contains the region of the BX-C from about iab-4 to the Abd-B m promoter. The overall structure of this BAC was verified by restriction enzyme mapping [using three restriction digests (EcoRI, XmaI, BamHI) (data not shown)].

### Abd-B Gal4 reporter BAC

Using *iab4-SacBII BACR24L18D* as a base we used recombineering to replace the start codon of the *Abd-Bm* isoform with sequence encoding the Gal4 transcription factor. First, a negative/positive selection cassette was created using the SacB gene and the Ampicillin resistance (AmpR) gene. SacBII was digested out of the plasmid pSK2-SACBK MAR using BamHI and EcoRI and cloned in pHSS7 [81]. The AmpR gene was then amplified with primers (*Amp BamHI* and *Amp XmaI*) carrying a BamHI and XmaI site. The amplified AmpR fragment was digested with BamHI and XmaI and cloned into a pHSS7-SacBII digested with the same enzymes, producing pHSS7-SacBII/Amp.

The SacBII/Amp cassette was flanked by two large homology regions using an overlap PCR strategy, as follows: The primers NHR L frw NotI/long and NHR L rev OL/long were first used to amplify the region 39201 bp to 40325 bp of iab4-SacBII BACR24L18D. At the same time, the primers NHR R frw OL/long and NHR R rev XmaI/long were used to amplify the 40326 to 41590 bp region of *iab4*-SacBII BACR24L18D. The two reaction products contain a 27 bp region of complementarity to mediate overlap PCR. Thus, the two fragments were mixed together with the primers NHR L frw NotI/long and NHR R rev XmaI/long in an overlap PCR reaction. The resulting fragment, containing the two homology domains fused together, was digested with NotI and XmaI and cloned into pHSS7 (pHSS7-NHR-L/NHR-R). Next, from the previously created pHSS7-SacBII/Amp, the double selection cassette was amplified with primers *NSx/A new F EcoRI* and *NCSx/A new R XbaI*. Digesting this PCR fragment with EcoRI and XbaI produced a fragment that could be inserted between the two homology domains of pHSS7-NHR-L/NHR-R creating pHSS7-NHR-L/SacBII-Amp/NHR-R.

The primers *F300 NS/A rec* and *R300 NS/A rec* were used to amplify a fragment from pHSS7-NHR-L/SacBII-Amp/NHR-R for recombineering. This fragment contained 300 bp of the homology regions on both sides of the cassette carrying the SacBII and AmpR genes. Once again recombineering was performed using the protocol of Soren Warming [80], where the target BAC was *iab4-SacBII BACR24L18D*. BAC DNA purified from the resulting ampicillin resistant/sucrose sensitive colonies were verified by extensive restriction enzyme digests. The new BAC was named *iab4-SacBII BACR24L18D N S/A ins*.

To replace the SacBII/AmpR cassette, the Gal4 gene (with a synthetic polyA tail) was PCR amplified from the plasmid pTnT Gal4 (unpublished, pTNT base vector from *Promega Corp.*, Madison, Wisconsin, USA) using the following primers: HR-R Gal4rep and HR-L Gal4rep. These primers contain 55 bp homology regions to mediate recombineering to the BX-C sequences just flanking the SacBII/AmpR cassette. The resulting targeting fragment was then phosphorylated by T4 kinase in order to improve the recombination reactions. After standard preparation of the recombineering DY380 strain containing *iab4-SacBII BACR24L18D N S/A ins*, bacterial colonies were selected on LB agar plates containing 10% sucrose. Restriction digestion of the candidate colonies with BamHI was performed in order to confirm the correct replacement of the SacBII/Amp cassette with Gal4.

### Injections of BACs

Using the PhiC31 system ([45]; www.flyc31.org), site 51C on the second chromosome was chosen for integration of the BACs into the fly genome. For better integration frequencies, all BACs were isolated on the day of injection using the NucleoBond PC 20 (Macherey-Nagel ref 740571) miniprep kit and resuspended in injection buffer [82]. Embryos were injected with BAC DNA (at about 50–100 ng/ul) through the chorion using the Eppendorf system (FemtoJet & TransferMan NK 2) equipped with Femtotips II glass needles. Integration efficiency was about 5%, based on the total number of fertile adults that yielded at least one integrant.

### Creation of a specific secondary cell Gal4 driver based on the cocu enhancer

The 2.8 kb putative enhancer sequence removed in *iab-6^cocu^* was amplified by PCR using primers D5 F and D5 R. Both of these primers contain a BamHI site at their 5′ ends. The amplified DNA fragment (called D5) was cloned into the BamHI site of the pChs-Gal4 plasmid, which contains a minimal Hsp70 promoter upstream of the coding sequence for Gal4 and the HSP70 3′UTR (Drosophila Genomics Resource Center [83]. Although clones with the enhancer in both orientations were isolated, we proceeded using a clone where the D5 R primer containing end was closest to Gal4. The D5-Gal4 cassette was then digested out of the pChs-Gal4 vector with NotI and cloned into the NotI site of pattB [45]. An insertion with the Gal4 coding sequence next to the *white* gene was selected for injection. This construct was integrated by Genetic Services Inc (Cambridge, Mass) into the VK00001 (59D3) platform [84]. The resulting integrant is named D5rsG4rs and referred as to D5-Gal4 in the text.

### Fly crosses and strains

All crosses were done using standard genetic techniques. *iab-7^Sz^*, *iab-6,7^IH^*, *iab-5,6^J82^*, and *iab-4,5,6^DB^* are described in [6]. The lines *iab-6^Δ5^* and *iab-6^4^* are described in [41]. The line *iab-6^Δ5^* was described as a deficiency without any phenotypic consequence. Following the LTR phenotype identified in this work the line was renamed *iab-6^cocu^* (reflecting that mates of these males fail to reject other suitors; “cocu” means “cuckold” in French). The BAC-*AbdB* ^Gal4^,*w^+^*, UAS-GFP/Cy line carrying the *Abd-B* Gal4 BAC reporter and a UAS-GFP marker on the second chromosome was created for this study by recombining a chromosome carrying the BAC and a UAS-GFP chromosome. The BAC reporter chromosome cannot exist as a homozygote. The 4.4E transgenic *lacZ* reporter line is described in [6]. The Gal4 expressing lines driven by a *paired* enhancer ((w^−^; prd-mf5.2,w^+^/CyO), (w^−^;prd-mf5.4,w^+^), (w^−^;prd-mf5.5,w^+^), (w^−^;prd-mf9.3,w^+^), (w^−^;prd-mf9.7,w^+^)) were obtained from Makus Noll's laboratory [46]. They were used in a cross with a UAS-*AbdBm* [85] flies for the experiment in which we tested for the ability of *Abd-Bm* to transform main cells into secondary cells.

All flies for fertility and fecundity assays, tests of receptivity and sperm competition, Western blotting, sperm counts, and PNGase F assays were raised at room temperature (23±1°C). Females were aged 3–5 days from eclosion in groups of 7–11 in glass vials on standard yeast-glucose media with added yeast. Males were aged 3–5 days from eclosion in groups of 10–20 in glass vials on standard yeast-glucose media.

### Antibody, X-Gal, and FM4–64 staining

Antibody and X-Gal staining on embryos and dissected accessory glands was performed as described in [12] and [42] respectively, using a 20 min fixation. The *Abd-B* primary antibody, obtained from the Developmental Studies Hybridoma Bank, was diluted 1∶4. Goat-anti-mouse secondary antibody, coupled to Alexa Fluor 488/555 X (Invitrogen AG), used to reveal *Abd-B* localization was used at 1∶500 dilution. Goat HRP coupled anti-mouse was obtained from Biorad and used at 1∶1'500 dilution. Staining with FM4–64 dye was done by placing a drop of the dye onto a microscope slide and placing a freshly dissected gland into it. The glands were immediately covered with a cover slip and visualized using fluorescent microscope at 555 nm.

### Fertility/fecundity, receptivity, sperm counts, Western blotting, and sperm competition assays

In all assays, we used 3–5 day old virgin females from a wild type strain (Canton-S for fertility/fecundity assays, receptivity assays, sperm counts, and for Western blotting experiments; and *cn bw* for sperm competition assays). Females were placed singly in glass vials with food and allowed access to an *iab-6^cocu^*, control male (heterozygous for the *iab-6^cocu^* mutation), DTA-E, or WT Canton-S male. Pairs were watched to confirm that mating had occurred. The male was removed upon dismounting. All statistical analysis was performed with the Jmp9 software.

In the fertility/fecundity assays, after mating, individual females were housed for 24 hours in glass vials on yeast-glucose media. After 24 hours each female was transferred to a fresh vial, and the eggs laid in the previous vial were counted. This process was repeated for a total of 10 days. Upon eclosion, all progeny from each vial were counted. Hatchability (# progeny/# eggs) was calculated per day and across the 10-day period for each female. Values greater than 1 represent instances where the number of progeny produced exceeded the number of eggs observed. This is an accurate representation of counter error, and was not normalized to 1. Small levels of counter error has a greater impact on hatchability for females that lay few eggs. Comparisons of egg and progeny production between control and experimental females were performed using a Wilcoxon non-parametric test and statistics comparing the overall 10 day trends were performed using a repeated measures ANOVA.

#### Receptivity assays

After mating, individual females were kept in a vial on yeast-glucose media for 1 day, 4 days, or 10 days after the start of mating (ASM). Each female was then moved to a fresh vial and provided with a single Canton S male. After addition of the single Canton S male, couples were observed at 15 min time intervals for one hour, and the proportion of successful matings was recorded. The original vials were kept to check for progeny from the first mating. Females that did not produce viable progeny were discarded from the assay. Comparisons between the remating frequency of control and experimental females were conducted using a one way ANOVA.

#### Sperm counts

After mating females were either frozen in liquid nitrogen at 2 h ASM or kept in glass vials on yeast-glucose media for 4 days and 10 days ASM and then frozen. The female reproductive tracts were removed and stained with orcein (as described in [33], [86], [87]. Sperm were visualized and counted using a transillumination microscope at 1000× magnification. Comparisons between the number of sperm present in control and experimental females were performed using a Wilcoxon non-parametric tests.

#### Sperm competition assays

After mating, individual females were housed for 3 days on yeast-glucose media. Females were then allowed access to a single *cn bw* male for 7 hours. Couples were observed for the first 4 hours in 15 min time intervals to determine the percent remating. After 7 hours the *cn bw* male was removed and the females were transferred individually to fresh vials and allowed to lay eggs for 4 days. They were then transferred individually to fresh food vials and allowed to lay eggs for an additional 4 days. Progeny were collected from each vial and assessed for the presence of red eyes (control or *iab-6^cocu^* male sire) or white eyes (*cn bw* sire). P1 was calculated as # progeny sired by the first male/total progeny. Comparisons between the P1 of control and experimental females were performed using a one way ANOVA and by Wilcoxon non-parametric tests.

#### Western blots

Females were frozen in liquid nitrogen at 15′, 30′, 1 h, and 2 h time points ASM and stored at −20°C until dissection. For later time points (1–7 days), females were kept individually on yeast-glucose media at room temperature before being frozen in liquid nitrogen and stored at −20°C until dissection. Males were also frozen in liquid nitrogen and stored at −20°C until dissection. Preparation of protein samples and Western blot analyses were performed as in [38], [39] except that gels in this study were 5–15% acrylamide gradient gels and were run at 100v for 1.5–2 hours. Due to size differences in organs across males and the lack of an optimal loading control for male accessory glands, all comparative samples contain an identical number of reproductive tracts (male or female).

PNGase F assays were performed using reagents from New England Biolabs Inc. Male accessory glands from 10 *iab-6^cocu^* males and 10 *iab-6^cocu^* heterozygous control males were dissected in 1×PBS and transferred to 10 ul 1× Glycoprotein Denaturing Buffer (GDB). Samples were ground and heated in GDB for 10 min at 100°C. Then 2 ul 10×G7 Reaction Buffer, 2 ul 10% NP40, and 4 ul ddH_2_O were added. Each sample was split into 9 ul aliquots. 1 ul PNGase F was added to one aliquot and 1 ul ddH_2_O was added to the other. All samples were then incubated for 1 hour at 37°C and frozen at −80°C overnight. 10 ul SDS sample buffer was added to each sample and then the samples were boiled for 5 min at 100°C. Western blots were performed as previously described except that 10.6% acrylamide gels were used and run at 40 v for 16 h to ensure adequate separation.

## Supporting Information

Figure S1 Rescue of *iab-6,7^IH^* by the *Abd-B* Bac. Panel A and B show male abdominal cuticle preparations from homozygous *iab-6,7^IH^* rescued with one copy of the *Abd-B* BAC (panel A) and homozygous *iab-6,7^IH^* (panel B). Male abdomens were cut along the dorsal midline and flattened on a slide. The dorsal surface of each abdominal segment has a rectangular plate of hard cuticle called the tergite. Only half of the tergites of the 4th, 5th and 6th abdominal segments (numbered) are visible, as well as the genitalia at the bottom. In as much as the *iab-6,7^IH^* phenotype is fully rescued by the *Abd-B* BAC the cuticle shown in panel A can be considered as wild type. Note that the 5th and 6th tergites are pigmented. The ventral surface of abdominal segments is composed of soft cuticle called the pleura. On the ventral midline of the pleura there are small plates of harder cuticle called sternites. In wild type (as well as in panel A), the 6th sternite, circled in red, can be easily distinguished from the more anterior sternites by its different shape and by the absence of bristles. Note also the absence of the 7^th^ abdominal segment present in embryos and larvae, which does not contribute to any adult structures after metamorphosis. B In *iab-6,7^IH^*, A6 is completely transformed into a copy of A5 as revealed by the presence of a 6^th^ sternite that completely resembles a more-anterior sternite, covered with bristles (circled in red in panel B). The striking appearance of a 7^th^ tergite is indicative of a homeotic transformation into A6. The transformation is however only partial as seen by the shape of the 7^th^ sternite that resembles the 6^th^, but harbors a few bristles (A5 character) see also reference [6].
(TIF)

Figure S2 Embyonic expression patterns driven by the *Abd-B-Gal4* Bac. Embryos were fixed and stained with antibodies directed against ß-galactosidase.(panels A,B,C and D) and *Abd-B* (panels E,F,G and H). Panel A,B and C show that the *Abd-B-Gal4* BAC mimics *Abd-B* temporal activation during germband elongation shown in panels F,G and H (see ref [89]). In panels D and E, stage 14 embryos were opened along the dorsal midline (through the amnioserosa) and flattened on a microscopic slide: anterior is at the top, the ventral midline with the developing CNS is visible in the center. Panel E is stained for *Abd-B*. The parasegmental boundaries are indicated. Arrows in panel D point towards the anterior, ectopic expression already visible earlier in panels B and C. Note also the group of neuroblasts expressing lacZ in parasegments anterior to PS10.
(TIF)

Figure S3 *Abd-B* mRNA expression in WT and in strains carrying the Bac with the *Abd-B* gene. Panels A through C show embryos through the process of germ band elongation hybridized with a strand-specific RNA probe to detect *Abd-B* expression. Like it is the case for *Abd-B* protein, RNA expression follows a temporal activation from posterior to anterior parasgements. A WT embryo at approximativey stage 15 is shown in panel D. At this stage *Abd-B* expression is restricted to the central nervous system. The parasegmental border are shown by acolades. Panel E show an embryo at the same stage from the strain that carries the BAC with the *Abd-B* gene. Oblic arrows show the sites of ectopic expression.
(TIF)

Figure S4 Larval and adult expression patterns driven by the *Abd-B-Gal4* BAC and endogenous *Abd-B* expression in wild type accessory gland and ejaculatory duct. A and B: pictures of live larvae and adult expressing GFP under the control of the *Abd-B-*Gal4 BAC. A third instar larva is shown in A. The arrow points towards the posterior part of the CNS that has fused with the brain after nerve chord contraction. The region corresponding to abdominal segments A5 to A8 is indicate by a bracket. An adult male is shown in panel B. Most of the fluorescence seen in the abdomen emanates from the accessory gland and the fat body. Panel C shows a WT pair of accessory gland connected with the ejaculatory duct stained with antibodies directed against *Abd-B*. Note the staining in the secondary cells at the tips of the accessory glands as well as the strong expression in the ejaculatory duct. Panels D, E and F show a magnification of the distal tip of an accessory gland stained with antibodies agains *Abd-B* (red) and with Dapi (blue). Note that both main and secondary cells are binucleated.
(TIF)

Figure S5 Rescue of *iab-6^cocu^* by an Abd-B expressing Bac. The top row shows photographs of the tip of accessory glands observed in “Nomarski” microscopy to visualize the characteristic vacuoles of the secondary cells (in panel A, a few secondary cells are circled). Note that these large vacuoles are lost in glands from *iab-6^cocu^* homozygotes (panel B). Panel C shows the secondary cells of a homozygous *iab-6^cocu^* gland carrying a copy of the *Abd-B* Bac on the 2^nd^ chromosome. Note the reapparance of a few vacuoles. This partial rescue suggests that a single copy of the *Abd-B* Bac does not resume the same level of *Abd-B* expression from the endogeneous locus. This partial rescue is paralleled by the fecundity and receptivity tests performed in females that were mated to *iab-6^cocu^* homozygous males carrying the *Abd-B* Bac (D and E). In panel D, the mean number of eggs laid per female after mating to either control males (blue line)), *iab-6^cocu^* males (red line), or BAC Rescue males (green line) over a 5 day period. Females mated to BAC Rescue males lay more eggs than females mated to *iab-6^cocu^* males (rmANOVA p = 0.0005*), though less than females mated to control males (rmANOVA Control:*iab-6^cocu^* p = <0.0001*, rmANOVA Control: BAC Rescue p = <0.0001*, Control N = 16, *iab-6^cocu^* N = 16, BAC Rescue N = 15) suggesting that rescue is only partial. B) Panel E depicts the percentage of mated females willing to mate within 1 hour of exposure to a wild type male at 4 days after an initial mating. Mates of BAC Rescue males are less receptive than mates of *iab-6^cocu^* males (WRST p = 0.0463*). However, both mates of *iab-6^cocu^* males and Rescue BAC males are more receptive than mates of control males (WRST Control: *iab-6^cocu^* p = <0.0001*, WRST Control: BAC Rescue p = <0.0006*, Control N = 41, *iab-6^cocu^* N = 48, BAC Rescue N = 38) further suggesting that rescue is not complete. Panel F Western blots of accessory gland extracts from control males (+lanes 1 and 4), *iab-6^cocu^* males (Δ lanes 2 and 3), and BAC Rescue males (R lanes 3, 6, and 7) probed with antibodies to CG1656. XR is a cross reactant band that always accompanies CG1656 and is included here as a loading control and also to control for abnormalities in gel mobility caused by sample prep or the gel itself. Each lane contains two pairs of AGs. Rescue of the glycosylation differences in *iab-6^cocu^* males is highly variable running the gammut of no rescue (lane 3), partial rescue (lane6), and full rescue (lane 7). The ability to rescue this apparent molecular weight difference suggests that Abd-B expression in the secondary cells is directly related to the glycosylation phenotype.
(TIF)

Figure S6 Phenocopying *iab-6^cocu^* phenotype by *Abd-B* RNAi. The top four panels A–D show secondary cells in which UAS-GFP is driven by the D5-Gal4 driver. In WT (A), the vacuoles are easily detectable as black discs in the background of GFP. In panel B, the D5 driver activates a UAS-Abd-B hairpin construct (obtained from the VDRC; [90]) to inactivate *Abd-B* by RNA interference (in addition to the UAS-GFP). Vacuoles are perhaps as numerous as in A, but overall smaller in size. In panel C, a UAS-Dicer was introduced to enhance RNA interference on top of the Abd-B hairpin construct and UAS-GFP. The GFP staining appears more uniform as a result a the much smaller size of the vacuoles. Panel D shows the uniform GFP staining in the background of *iab-6^cocu^*. Panels E depicts the egg laying counts from females mated to WT males (light and dark green lines), or males in which the D5-driver activates the *Abd-B* hairpin construct (blue line) or males *iab-6^cocu^*(brown line). There is no noticable difference in fertility between the WT control and the males in which the *Abd-B* hairpin construct alone is active. However a siginficant difference in egg laying is observed when RNA interference is enhanced by the introduction of a UAS Dicer (Panel F) in the genotypes mentioned above (as revealed by the difference between the green and blue lines). In panel F, the mean number of eggs laid per female mated to either control males (green lines), *iab-6^cocu^* males (red line), or *Abd-B^RNAi^ Dicer* males (blue line) over a 8 day period. Mates of *Abd-B^RNAi^ Dicer* males lay more eggs than mates of *iab-6^cocu^* males (rmANOVA p = <0.0001). However, both mates of *iab-6^cocu^* males and *Abd-B^RNAi^ Dicer* males lay fewer eggs than mates of control males (rmANOVA Control 1(dark green line) & 2(light green line) :*iab-6^cocu^* p = <0.0001*, rmANOVA Control 1: *Abd-B^RNAi^ Dicer* p = 0.0069*, rmANOVA Control 2: *Abd-B^RNAi^ Dicer* p = <0.0001*, Control 1 N = 16, Control 2 N = 17, *iab-6^cocu^* N = 18, *Abd-B^RNAi^ Dicer* N = 17) suggesting that RNAi knockdown is not complete.
(TIF)

Figure S7 Mates of *iab-6^cocu^* heterozygous males show normal egg-laying behavior. The mean number of eggs laid per female mated to either Canton-S (dot dashed line), *iab-6^cocu^*/+ (Control) males (dashed line), *iab-6^cocu^* males (solid line), or DTA-E males (grey dot dashed line) over a 5 day period. Mates of *iab-6^cocu^* males lay fewer eggs over 5 days when compared to mates of either Canton-S (rmANOVA p = <0.0001) or *iab-6^cocu^*/+ (Control) males (rmANOVA p = <0.0001*, Canton-S N = 22, *iab-6^cocu^*/+ (Control) N = 16, *iab-6^cocu^* N = 20, DTA-E N = 24). There was no significant difference in egg-laying between Canton-S or *iab-6^cocu^*/+ controls (rmANOVA p = 0.531).
(TIF)

Figure S8 Secondary cell specific knockdown of seminal fluid proteins. Western blots using antibodies to CG1656, CG17575, and Acp62F. All lanes contain accessory gland extracts from two virgin males (control or RNAi). When driven by *tubulin*-GAL4, presence of each of the three UAS-AcpRNAi constructs shown in the figure (and CG1652, not shown) greatly reduced the amount of the targeted Acp compared to controls (T = *tubulin*-GAL4; + = control, − = RNAi). In contrast, when we used the D1rsG4rs driver to drive expression in the secondary cells of the male accessory gland CG17575 and CG1656 were knocked down (as was CG1652, not shown) but levels of Acp62F, a main cell-expressed Acp [51], were not affected (D1 = D1rsG4rs driver; + = control, − = RNAi).
(TIF)

Figure S9 Quantification of signals from Figure 4C, 4D and Figure 7B. Normalization of the integrated optical density (IOD) of SP (Figure 4C and 4D) and CG17575 (Figure 7B) signals on Western blots of protein from reproductive tracts of mated females. Signals were normalized to the IOD of tubulin signals on the same blots. IOD was determined using Image-J. Because tubulin signal in protein extracts from male accessory glands is highly variable (JLS and MFW unpublished), amounts of SP or CG17575 for accessory gland samples were not normalized to tubulin signals and are not graphed here; each extract contains 1 pair of accessory glands A) Normalized IOD of SP from Figure 4C. Mates of *iab-6^cocu^* males have less SP in their reproductive tracts than controls at all long-term storage time points (1 d, 4 d, and 7 d ASM). A cosmetic defect in the tubulin bands and oversaturation of SP at the 2 h time point make interpreting the normalization results at 2 h difficult (see part B). B) Normalized IOD of SP from Figure 4D, 2 h ASM. Mates of *iab-6^cocu^* males store less SP in the seminal receptacle (SR) at 2 h ASM (20 SRs per sample). Average SP/total RT+SR per female is calculated by averaging the SP signals for the dilution series and dividing by the averaged tubulin signals for those series. The average SR signal per female was then combined with this value. C) Normalized IOD of CG17575 from Figure 7B. Mates of *iab-6^cocu^* males have less SP stored in their reproductive tracts than controls at all time points (15′, 30′, and 1 h ASM) and especially at 15′ and 1 h ASM.
(TIF)

Text S1 Evidences that BAC *Abd-B* driven expression resumes endogenous *Abd-B* expression and additional technical informations
(DOCX)
