# Supplementary figures and images for: A Novel Function for the Hox Gene Abd-B in the Male Accessory Gland Regulates the Long-Term Female Post-Mating Response in Drosophila
Source: PLoS Genet. 2013 Mar 28;9(3):e1003395. doi: 10.1371/journal.pgen.1003395 (PMC3610936; doi:10.1371/journal.pgen.1003395)

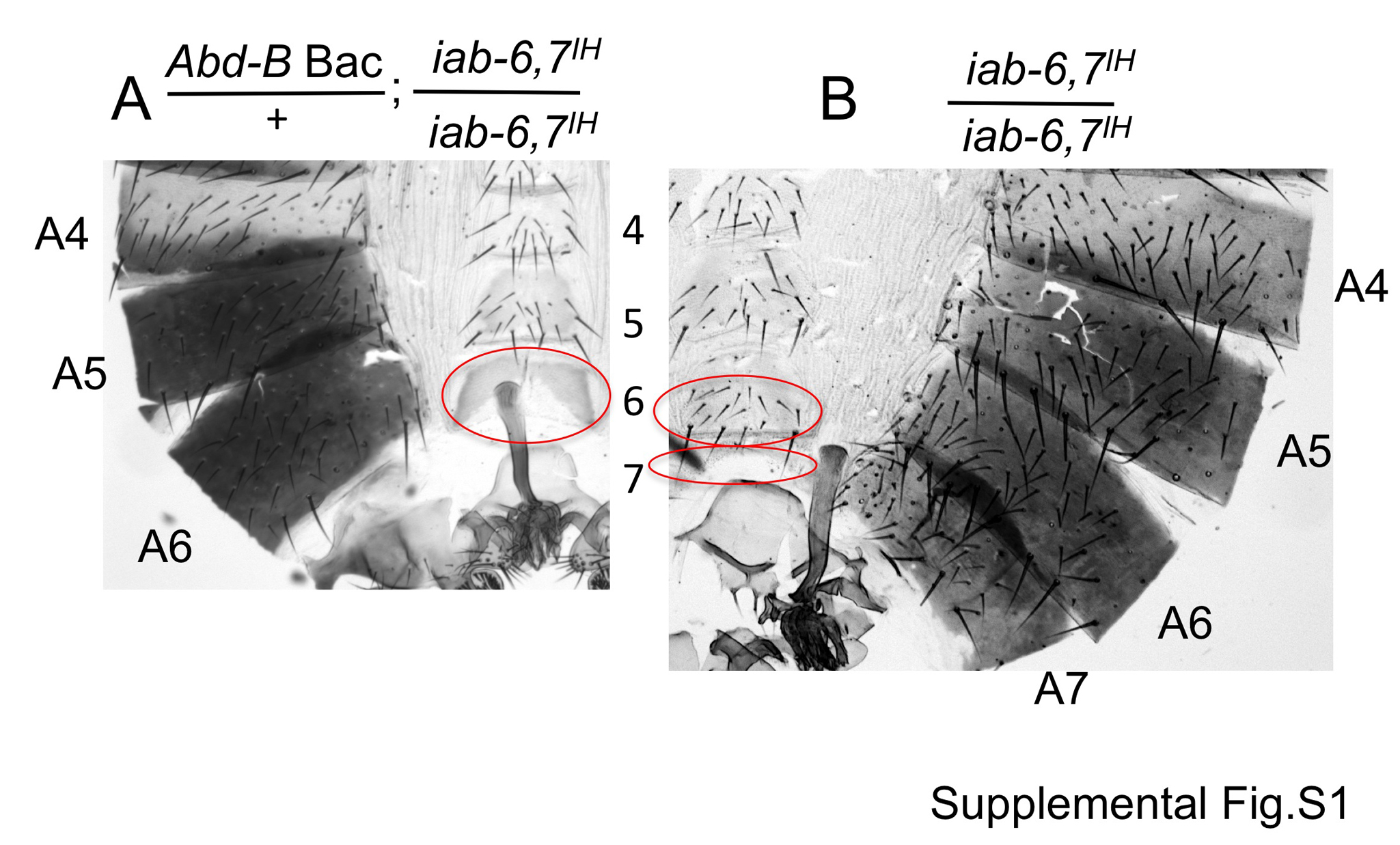

Supplement: Figure S1 — Rescue of iab-6,7IH by the Abd-B Bac. Panel A and B show male abdominal cuticle preparations from homozygous iab-6,7IH rescued with one copy of the Abd-B BAC (panel A) and homozygous iab-6,7IH (panel B). Male abdomens were cut along the dorsal midline and flattened on a slide. The dorsal surface of each abdominal segment has a rectangular plate of hard cuticle called the tergite. Only half of the tergites of the 4th, 5th and 6th abdominal segments (numbered) are visible, as well as the genitalia at the bottom. In as much as the iab-6,7IH phenotype is fully rescued by the Abd-B BAC the cuticle shown in panel A can be considered as wild type. Note that the 5th and 6th tergites are pigmented. The ventral surface of abdominal segments is composed of soft cuticle called the pleura. On the ventral midline of the pleura there are small plates of harder cuticle called sternites. In wild type (as well as in panel A), the 6th sternite, circled in red, can be easily distinguished from the more anterior sternites by its different shape and by the absence of bristles. Note also the absence of the 7th abdominal segment present in embryos and larvae, which does not contribute to any adult structures after metamorphosis. B In iab-6,7IH, A6 is completely transformed into a copy of A5 as revealed by the presence of a 6th sternite that completely resembles a more-anterior sternite, covered with bristles (circled in red in panel B). The striking appearance of a 7th tergite is indicative of a homeotic transformation into A6. The transformation is however only partial as seen by the shape of the 7th sternite that resembles the 6th, but harbors a few bristles (A5 character) see also reference [6]. (TIF) [file pgen.1003395.s001.tif]

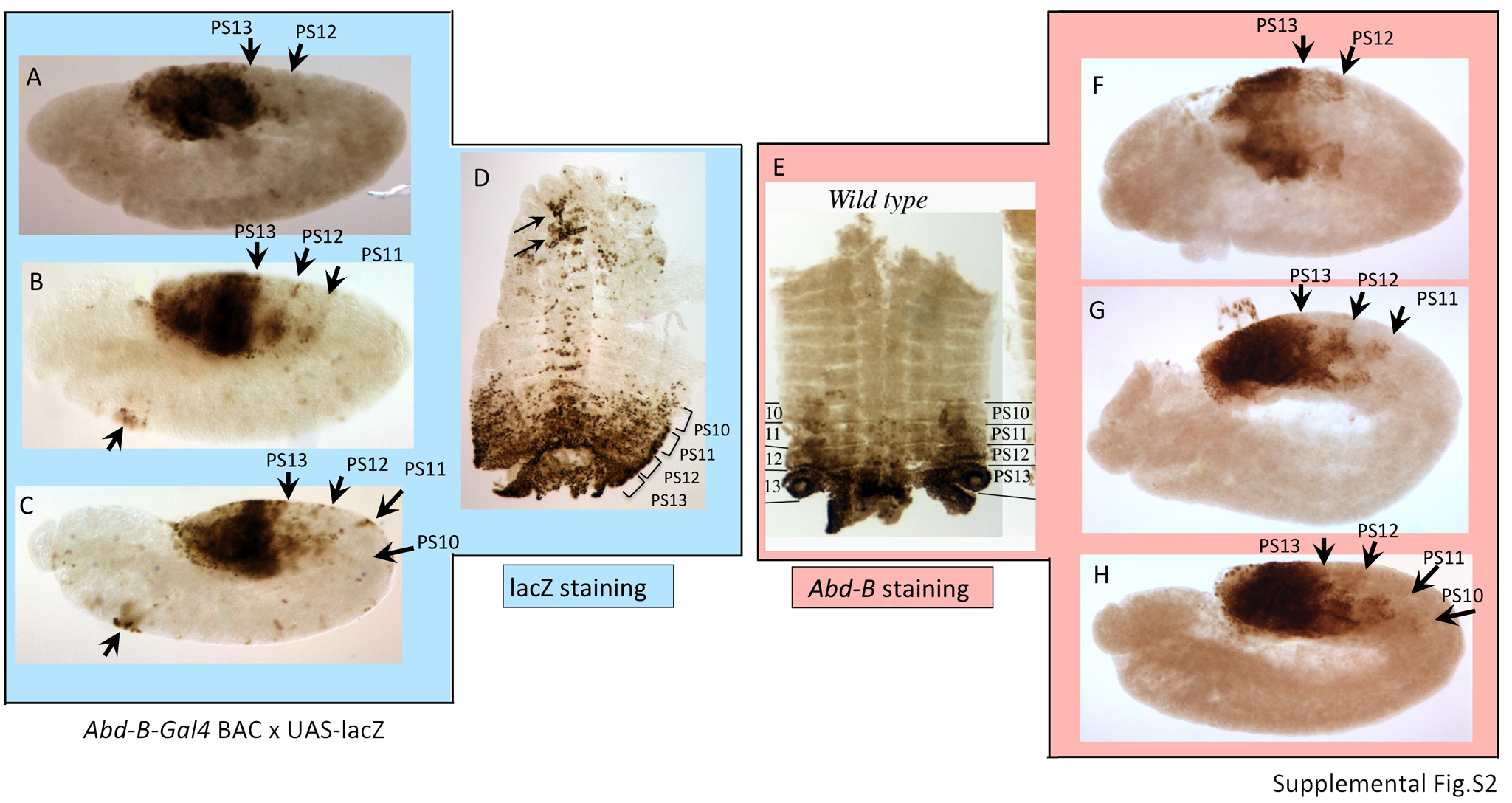

Supplement: Figure S2 — Embyonic expression patterns driven by the Abd-B-Gal4 Bac. Embryos were fixed and stained with antibodies directed against ß-galactosidase.(panels A,B,C and D) and Abd-B (panels E,F,G and H). Panel A,B and C show that the Abd-B-Gal4 BAC mimics Abd-B temporal activation during germband elongation shown in panels F,G and H (see ref [89]). In panels D and E, stage 14 embryos were opened along the dorsal midline (through the amnioserosa) and flattened on a microscopic slide: anterior is at the top, the ventral midline with the developing CNS is visible in the center. Panel E is stained for Abd-B. The parasegmental boundaries are indicated. Arrows in panel D point towards the anterior, ectopic expression already visible earlier in panels B and C. Note also the group of neuroblasts expressing lacZ in parasegments anterior to PS10. (TIF) [file pgen.1003395.s002.tif]

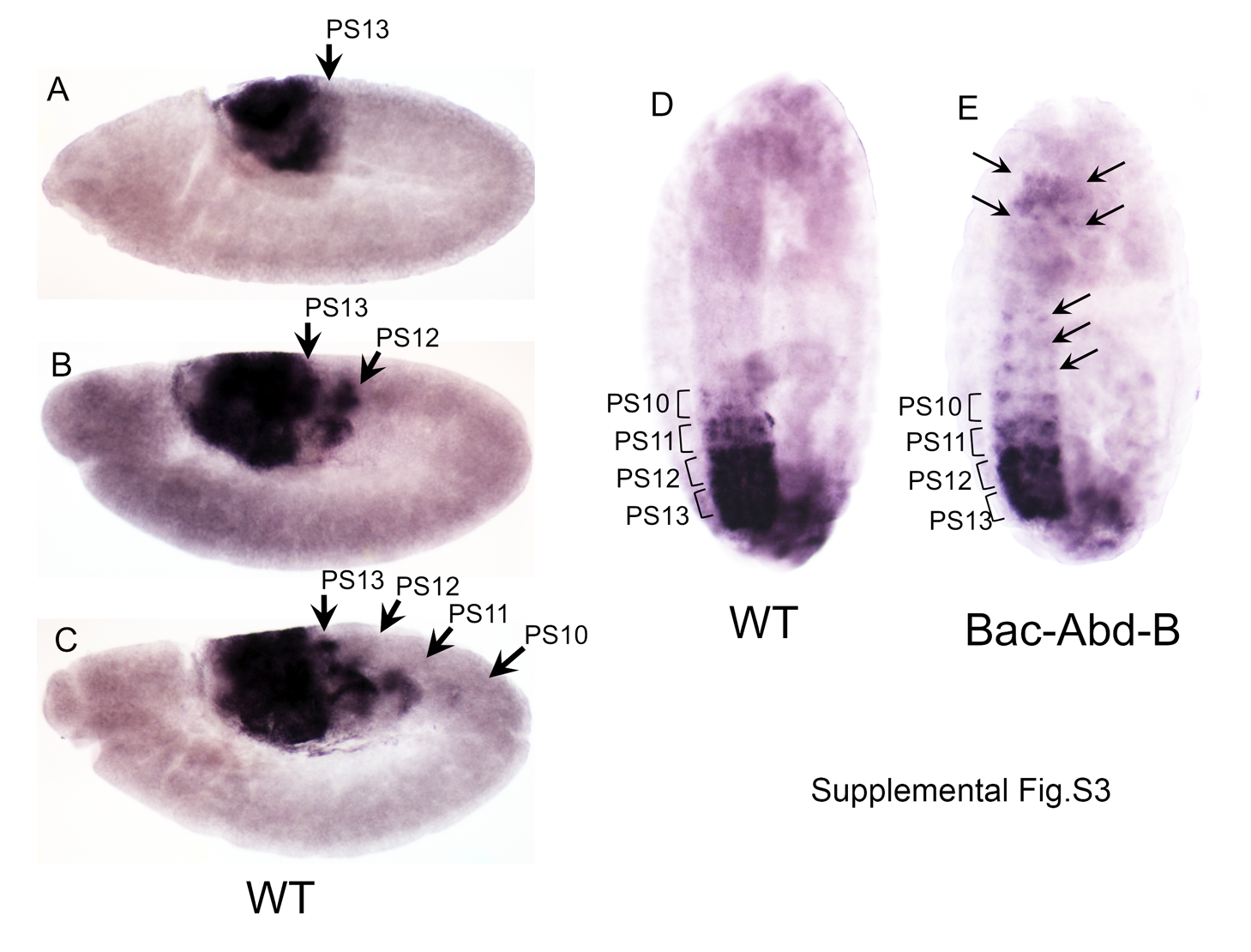

Supplement: Figure S3 — Abd-B mRNA expression in WT and in strains carrying the Bac with the Abd-B gene. Panels A through C show embryos through the process of germ band elongation hybridized with a strand-specific RNA probe to detect Abd-B expression. Like it is the case for Abd-B protein, RNA expression follows a temporal activation from posterior to anterior parasgements. A WT embryo at approximativey stage 15 is shown in panel D. At this stage Abd-B expression is restricted to the central nervous system. The parasegmental border are shown by acolades. Panel E show an embryo at the same stage from the strain that carries the BAC with the Abd-B gene. Oblic arrows show the sites of ectopic expression. (TIF) [file pgen.1003395.s003.tif]

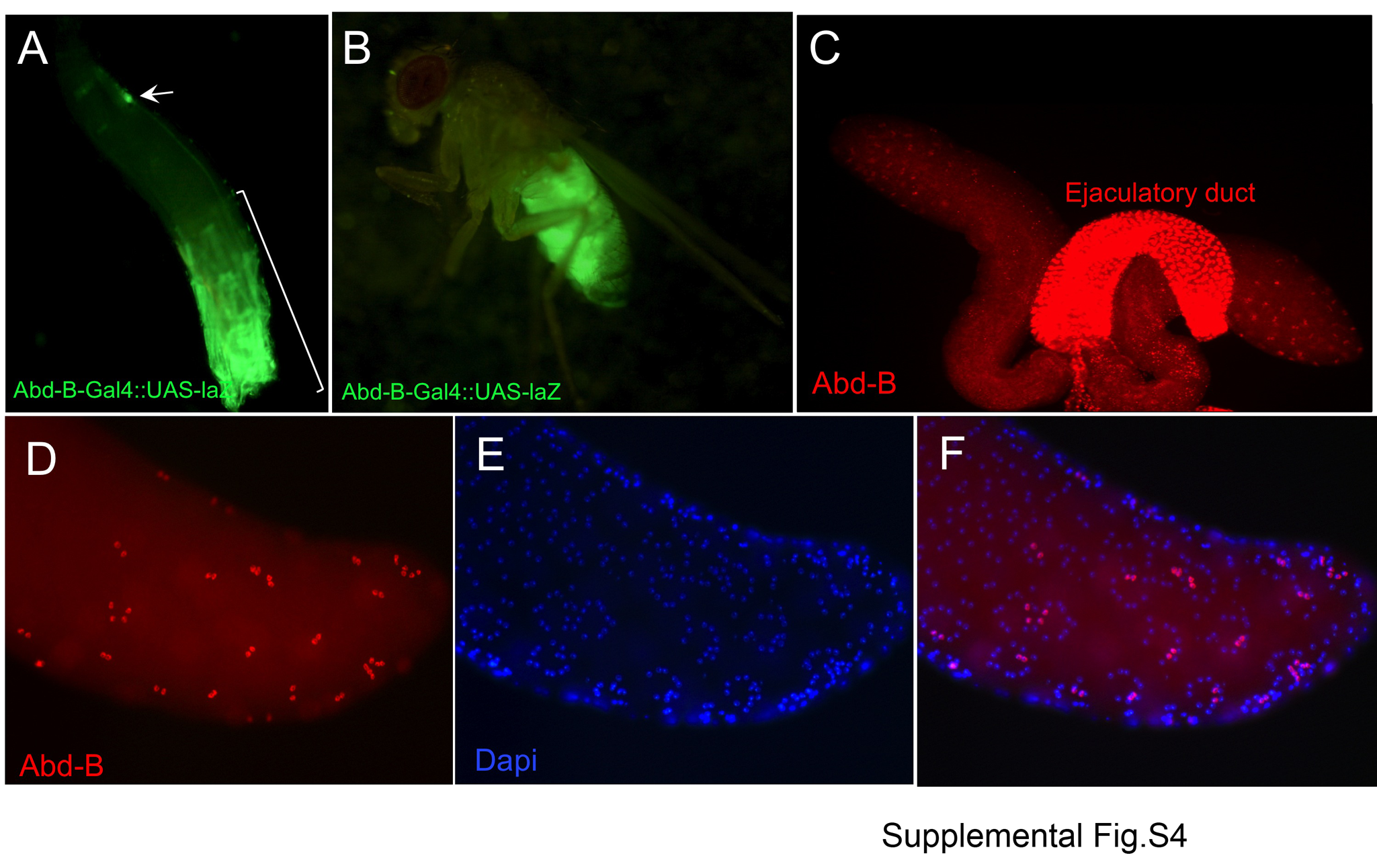

Supplement: Figure S4 — Larval and adult expression patterns driven by the Abd-B-Gal4 BAC and endogenous Abd-B expression in wild type accessory gland and ejaculatory duct. A and B: pictures of live larvae and adult expressing GFP under the control of the Abd-B-Gal4 BAC. A third instar larva is shown in A. The arrow points towards the posterior part of the CNS that has fused with the brain after nerve chord contraction. The region corresponding to abdominal segments A5 to A8 is indicate by a bracket. An adult male is shown in panel B. Most of the fluorescence seen in the abdomen emanates from the accessory gland and the fat body. Panel C shows a WT pair of accessory gland connected with the ejaculatory duct stained with antibodies directed against Abd-B. Note the staining in the secondary cells at the tips of the accessory glands as well as the strong expression in the ejaculatory duct. Panels D, E and F show a magnification of the distal tip of an accessory gland stained with antibodies agains Abd-B (red) and with Dapi (blue). Note that both main and secondary cells are binucleated. (TIF) [file pgen.1003395.s004.tif]

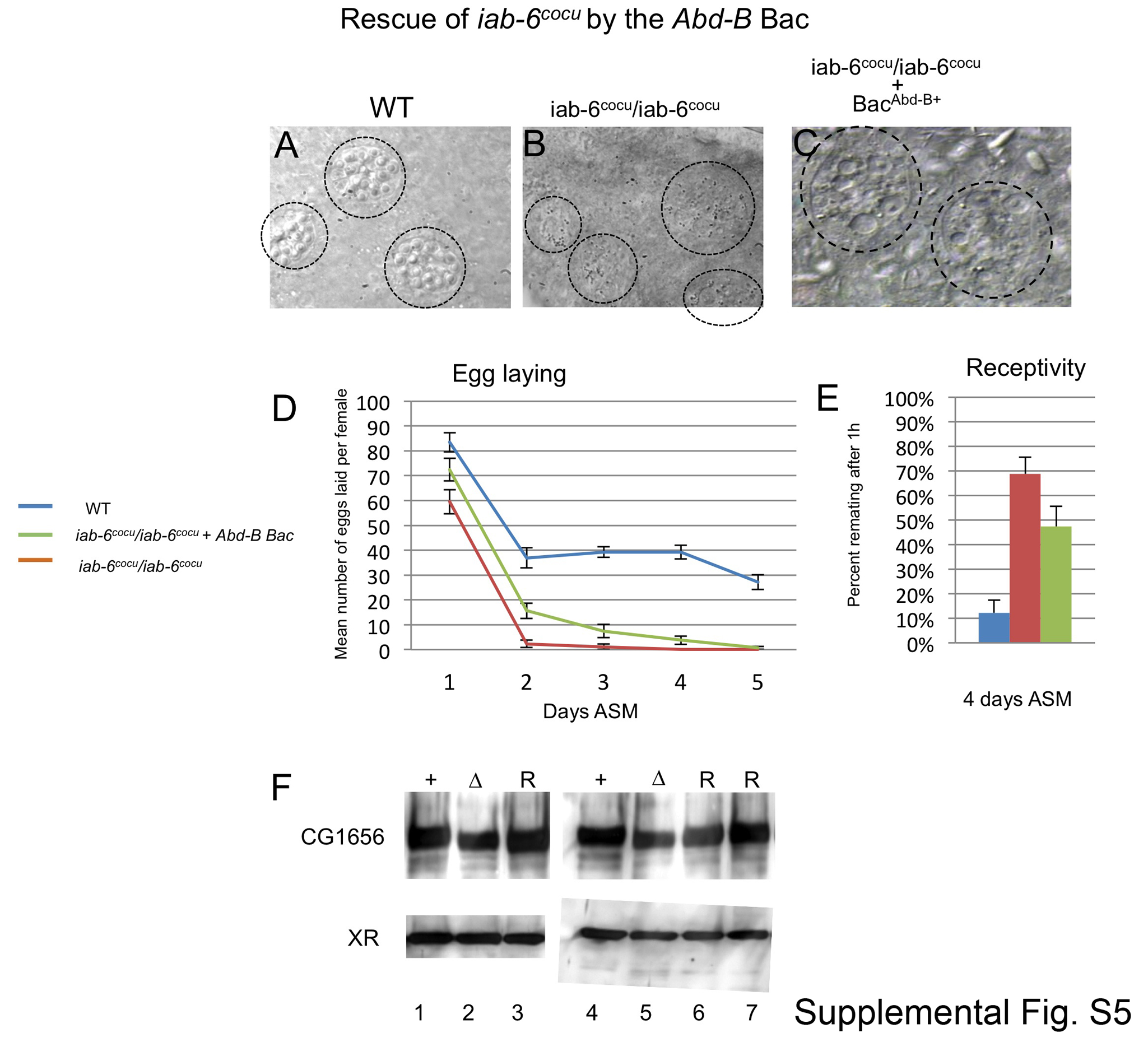

Supplement: Figure S5 — Rescue of iab-6cocu by an Abd-B expressing Bac. The top row shows photographs of the tip of accessory glands observed in “Nomarski” microscopy to visualize the characteristic vacuoles of the secondary cells (in panel A, a few secondary cells are circled). Note that these large vacuoles are lost in glands from iab-6cocu homozygotes (panel B). Panel C shows the secondary cells of a homozygous iab-6cocu gland carrying a copy of the Abd-B Bac on the 2nd chromosome. Note the reapparance of a few vacuoles. This partial rescue suggests that a single copy of the Abd-B Bac does not resume the same level of Abd-B expression from the endogeneous locus. This partial rescue is paralleled by the fecundity and receptivity tests performed in females that were mated to iab-6cocu homozygous males carrying the Abd-B Bac (D and E). In panel D, the mean number of eggs laid per female after mating to either control males (blue line)), iab-6cocu males (red line), or BAC Rescue males (green line) over a 5 day period. Females mated to BAC Rescue males lay more eggs than females mated to iab-6cocu males (rmANOVA p = 0.0005*), though less than females mated to control males (rmANOVA Control:iab-6cocu p = <0.0001*, rmANOVA Control: BAC Rescue p = <0.0001*, Control N = 16, iab-6cocu N = 16, BAC Rescue N = 15) suggesting that rescue is only partial. B) Panel E depicts the percentage of mated females willing to mate within 1 hour of exposure to a wild type male at 4 days after an initial mating. Mates of BAC Rescue males are less receptive than mates of iab-6cocu males (WRST p = 0.0463*). However, both mates of iab-6cocu males and Rescue BAC males are more receptive than mates of control males (WRST Control: iab-6cocu p = <0.0001*, WRST Control: BAC Rescue p = <0.0006*, Control N = 41, iab-6cocu N = 48, BAC Rescue N = 38) further suggesting that rescue is not complete. Panel F Western blots of accessory gland extracts from control males (+lanes 1 and 4), iab-6cocu males (Δ lanes 2 and 3), and BAC [file pgen.1003395.s005.tif]

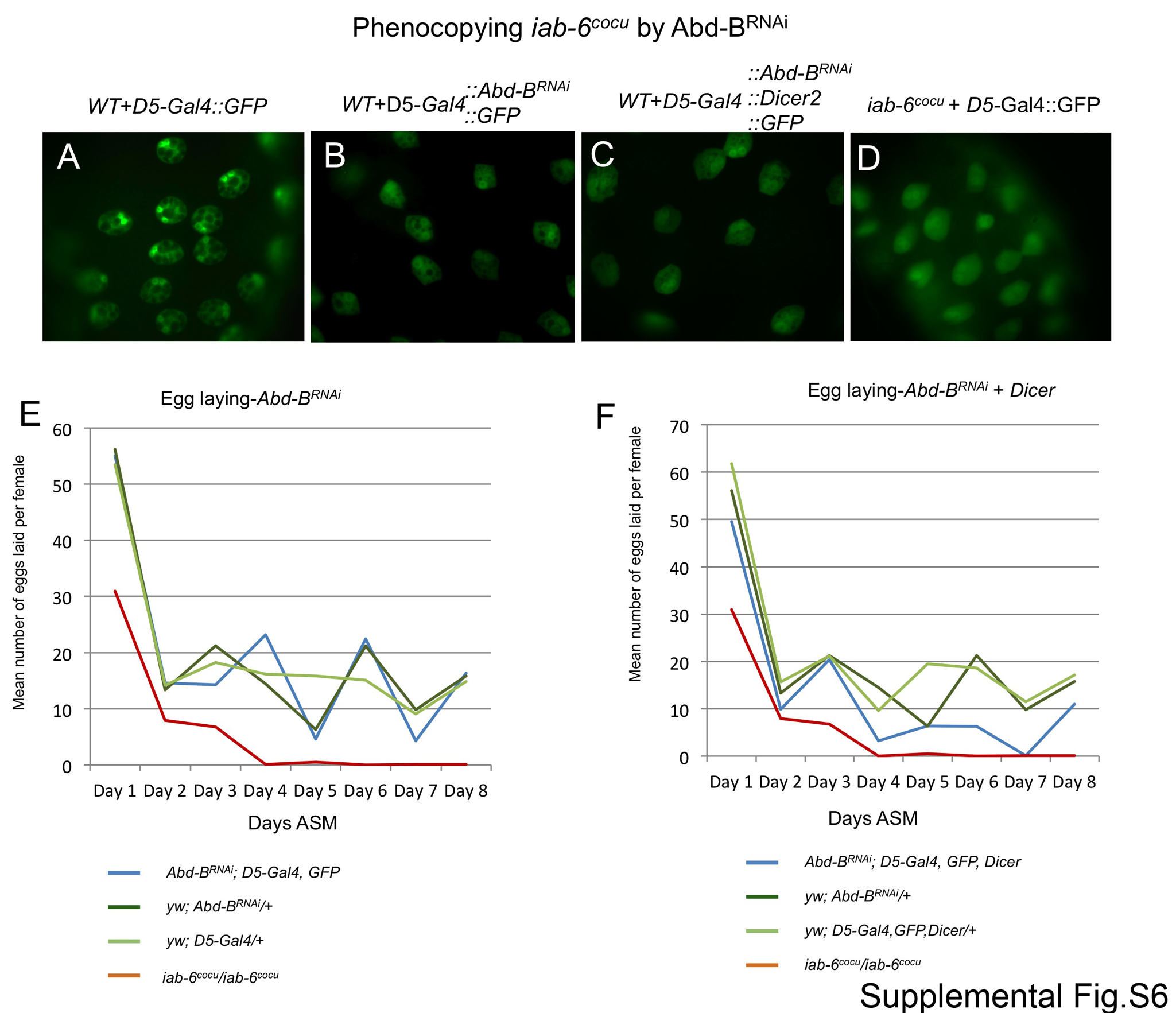

Supplement: Figure S6 — Phenocopying iab-6cocu phenotype by Abd-B RNAi. The top four panels A–D show secondary cells in which UAS-GFP is driven by the D5-Gal4 driver. In WT (A), the vacuoles are easily detectable as black discs in the background of GFP. In panel B, the D5 driver activates a UAS-Abd-B hairpin construct (obtained from the VDRC; [90]) to inactivate Abd-B by RNA interference (in addition to the UAS-GFP). Vacuoles are perhaps as numerous as in A, but overall smaller in size. In panel C, a UAS-Dicer was introduced to enhance RNA interference on top of the Abd-B hairpin construct and UAS-GFP. The GFP staining appears more uniform as a result a the much smaller size of the vacuoles. Panel D shows the uniform GFP staining in the background of iab-6cocu. Panels E depicts the egg laying counts from females mated to WT males (light and dark green lines), or males in which the D5-driver activates the Abd-B hairpin construct (blue line) or males iab-6cocu(brown line). There is no noticable difference in fertility between the WT control and the males in which the Abd-B hairpin construct alone is active. However a siginficant difference in egg laying is observed when RNA interference is enhanced by the introduction of a UAS Dicer (Panel F) in the genotypes mentioned above (as revealed by the difference between the green and blue lines). In panel F, the mean number of eggs laid per female mated to either control males (green lines), iab-6cocu males (red line), or Abd-BRNAi Dicer males (blue line) over a 8 day period. Mates of Abd-BRNAi Dicer males lay more eggs than mates of iab-6cocu males (rmANOVA p = <0.0001). However, both mates of iab-6cocu males and Abd-BRNAi Dicer males lay fewer eggs than mates of control males (rmANOVA Control 1(dark green line) & 2(light green line) :iab-6cocu p = <0.0001*, rmANOVA Control 1: Abd-BRNAi Dicer p = 0.0069*, rmANOVA Control 2: Abd-BRNAi Dicer p = <0.0001*, Control 1 N = 16, Control 2 N = 17, iab-6cocu N = 18, Abd-BRNAi Dicer N = 17) suggesting that R [file pgen.1003395.s006.tif]

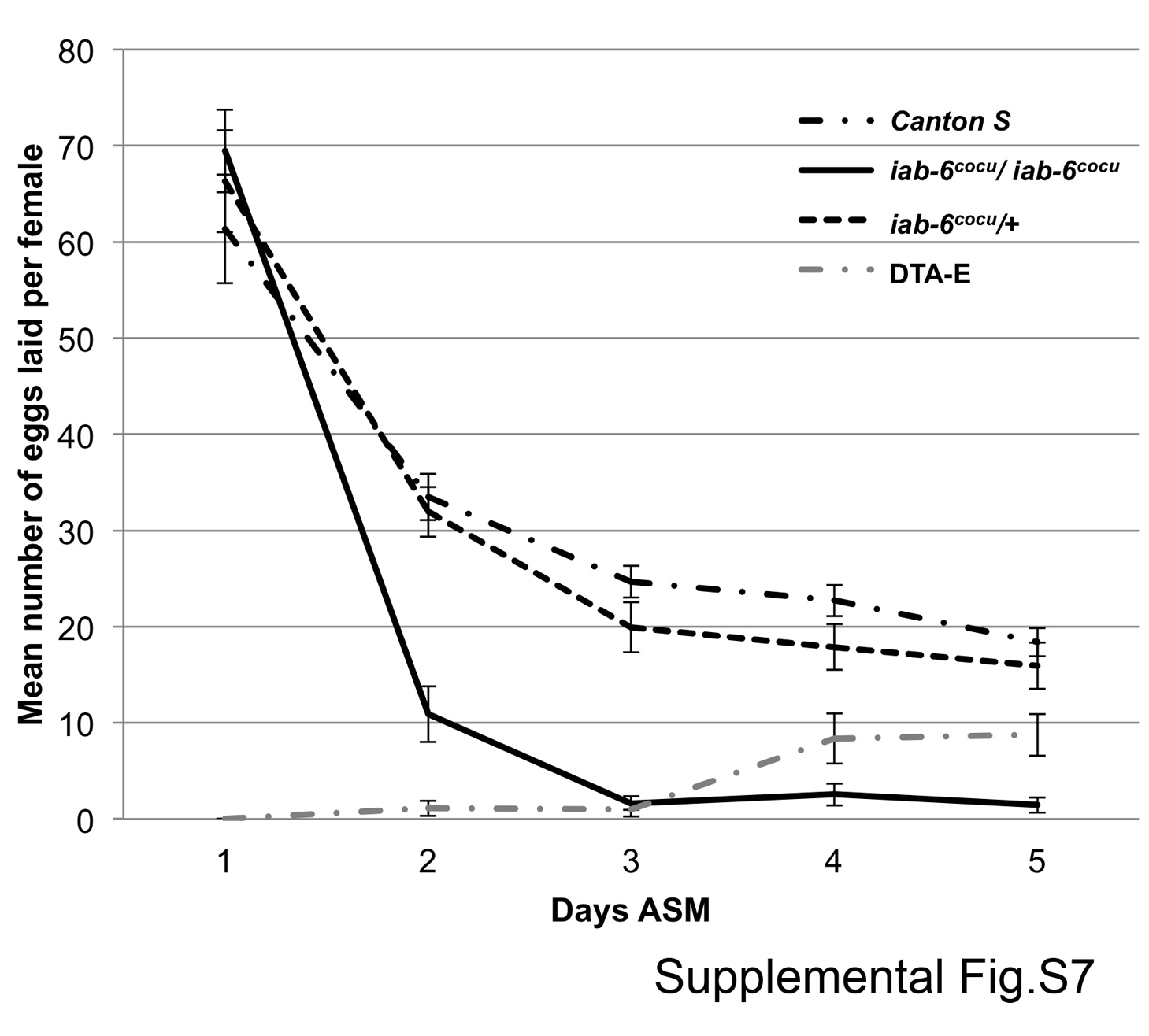

Supplement: Figure S7 — Mates of iab-6cocu heterozygous males show normal egg-laying behavior. The mean number of eggs laid per female mated to either Canton-S (dot dashed line), iab-6cocu/+ (Control) males (dashed line), iab-6cocu males (solid line), or DTA-E males (grey dot dashed line) over a 5 day period. Mates of iab-6cocu males lay fewer eggs over 5 days when compared to mates of either Canton-S (rmANOVA p = <0.0001) or iab-6cocu/+ (Control) males (rmANOVA p = <0.0001*, Canton-S N = 22, iab-6cocu/+ (Control) N = 16, iab-6cocu N = 20, DTA-E N = 24). There was no significant difference in egg-laying between Canton-S or iab-6cocu/+ controls (rmANOVA p = 0.531). (TIF) [file pgen.1003395.s007.tif]

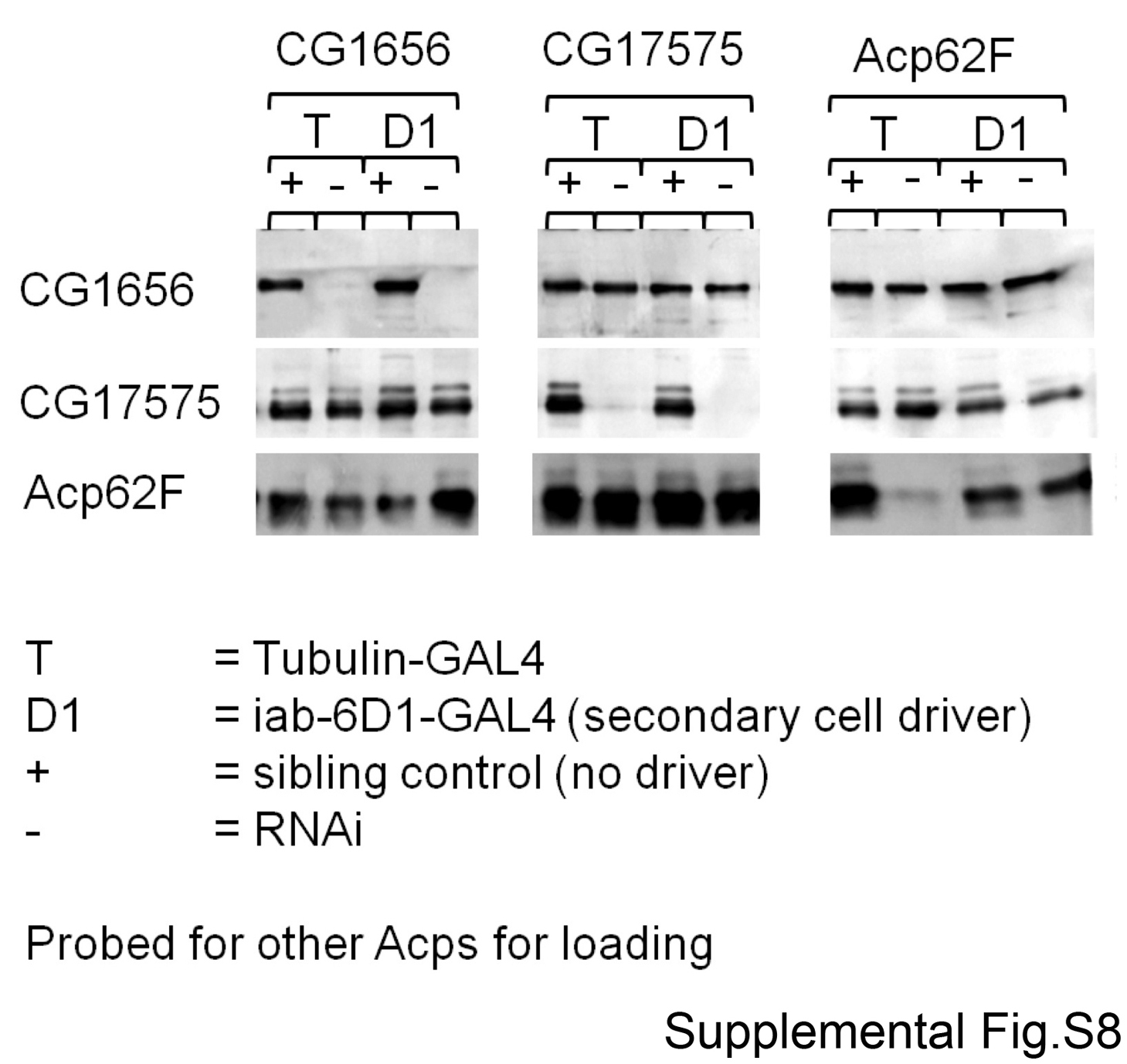

Supplement: Figure S8 — Secondary cell specific knockdown of seminal fluid proteins. Western blots using antibodies to CG1656, CG17575, and Acp62F. All lanes contain accessory gland extracts from two virgin males (control or RNAi). When driven by tubulin-GAL4, presence of each of the three UAS-AcpRNAi constructs shown in the figure (and CG1652, not shown) greatly reduced the amount of the targeted Acp compared to controls (T = tubulin-GAL4; + = control, − = RNAi). In contrast, when we used the D1rsG4rs driver to drive expression in the secondary cells of the male accessory gland CG17575 and CG1656 were knocked down (as was CG1652, not shown) but levels of Acp62F, a main cell-expressed Acp [51], were not affected (D1 = D1rsG4rs driver; + = control, − = RNAi). (TIF) [file pgen.1003395.s008.tif]

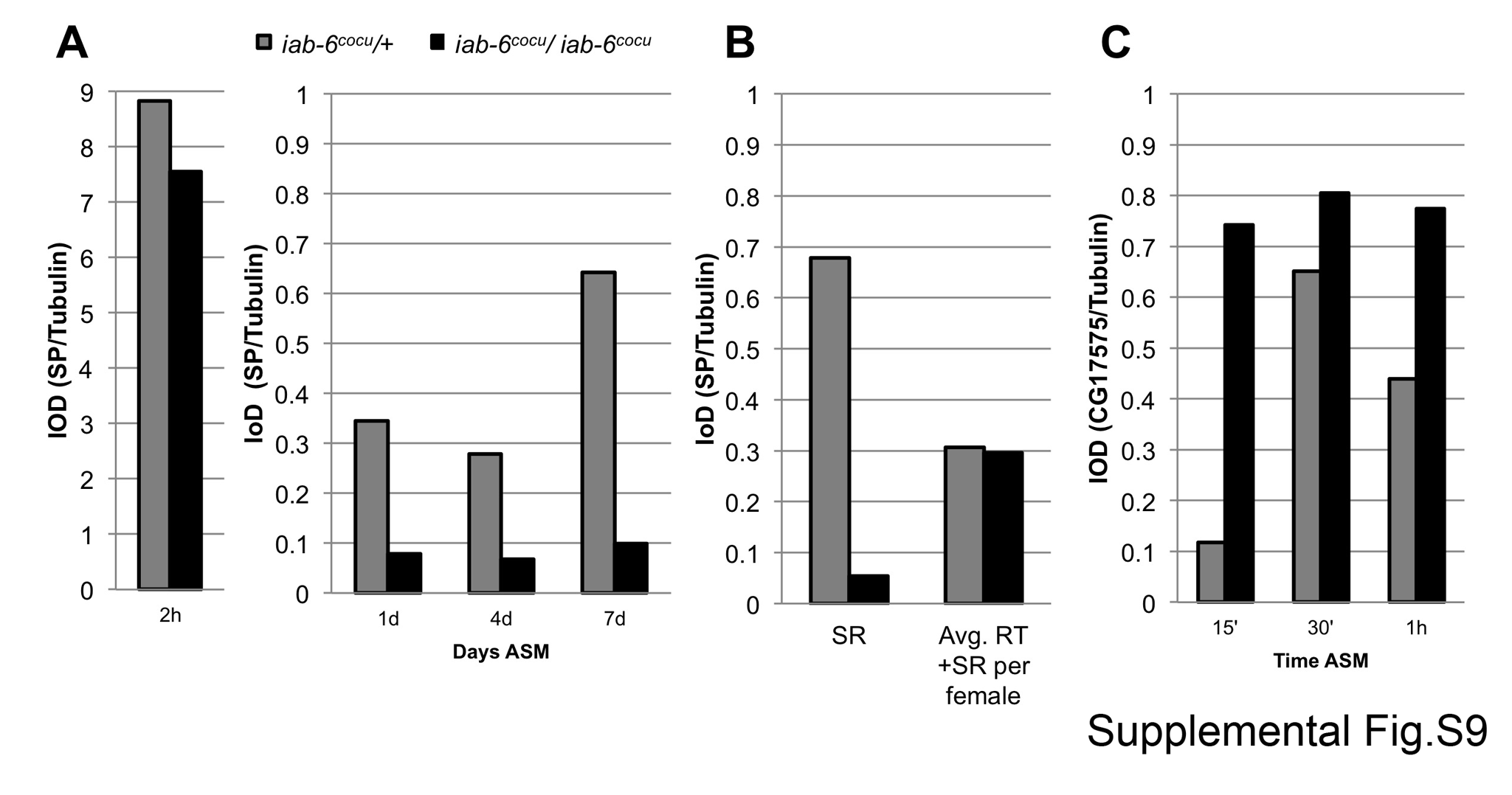

Supplement: Figure S9 — Quantification of signals from Figure 4C, 4D and Figure 7B. Normalization of the integrated optical density (IOD) of SP (Figure 4C and 4D) and CG17575 (Figure 7B) signals on Western blots of protein from reproductive tracts of mated females. Signals were normalized to the IOD of tubulin signals on the same blots. IOD was determined using Image-J. Because tubulin signal in protein extracts from male accessory glands is highly variable (JLS and MFW unpublished), amounts of SP or CG17575 for accessory gland samples were not normalized to tubulin signals and are not graphed here; each extract contains 1 pair of accessory glands A) Normalized IOD of SP from Figure 4C. Mates of iab-6cocu males have less SP in their reproductive tracts than controls at all long-term storage time points (1 d, 4 d, and 7 d ASM). A cosmetic defect in the tubulin bands and oversaturation of SP at the 2 h time point make interpreting the normalization results at 2 h difficult (see part B). B) Normalized IOD of SP from Figure 4D, 2 h ASM. Mates of iab-6cocu males store less SP in the seminal receptacle (SR) at 2 h ASM (20 SRs per sample). Average SP/total RT+SR per female is calculated by averaging the SP signals for the dilution series and dividing by the averaged tubulin signals for those series. The average SR signal per female was then combined with this value. C) Normalized IOD of CG17575 from Figure 7B. Mates of iab-6cocu males have less SP stored in their reproductive tracts than controls at all time points (15′, 30′, and 1 h ASM) and especially at 15′ and 1 h ASM. (TIF) [file pgen.1003395.s009.tif]
