# Supplementary material for: A Novel Function for the Hox Gene Abd-B in the Male Accessory Gland Regulates the Long-Term Female Post-Mating Response in Drosophila
Source: PLoS Genet. 2013 Mar 28;9(3):e1003395. doi: 10.1371/journal.pgen.1003395 (PMC3610936; doi:10.1371/journal.pgen.1003395)
Supplement: Text S1 — Evidences that BAC Abd-B driven expression resumes endogenous Abd-B expression and additional technical informations (DOCX) [file pgen.1003395.s010.docx]

**SUPPORTING INFORMATIONS**

**Evidences that BAC *Abd-B* driven expression resumes endogenous *Abd-B* expression.** Genetic and expressions studies strongly support the statement that the sequences included within in the 108kb-long BAC properly regulate the extra copy of *Abd-B.* First, patterns and levels of *Abd-B* expression are critical for the normal development of the fly. For example, rearrangements breaks that split the BX-C between *abd-A* and *Abd-B* often cause ectopic expression of *Abd-B*; this is revealed by a dominant gain-of-function (GOF) phenotype, in which black pigmentation appears in abdominal segments anterior to the 5^th^ (A5) in males. The fact that the extra copy of *Abd-B* carried by the BAC is not associated with any such GOF phenotype indicates that the BAC does not mis-express *Abd-B* out of its normal realm of action in the epidermis. Second, the dose-sensitivity of *Abd-B* is also revealed by the fact that *Abd-B* is one of very few *Drosophila* loci that are haplo-sterile. In this respect, restoration of fertility in both sexes of *Abd-B* hemizygous flies by the insertion of the *Abd-B* BAC reinforces the idea that the BAC achieves a reasonable level of *Abd-B* expression. Third, we find that the presence of a copy of the BAC rescues the mutant phenotypes of the *iab-6,7^IH^* and *iab-5,6^J82^* deficiencies (see Supplemental Fig.S1).

These genetic evidences are paralleled by expression studies. First, we observe a fairly normal *Abd-B* expression pattern by antibody staining of embryos carrying the wild-type *Abd-B* BAC, confirming the absence of ectopic expression To follow the expression pattern driven by the BAC alone, we crossed the Abd-B-Gal4 BAC derivative to flies carrying a UAS-lacZ reporter and performed staining with antibodies directed against ß-galactosidase. The embryonic lacZ expression patterns observed in Fig. S2 (panels A-D) resemble normal *Abd-B* expression. This is illustrated by the temporal activation in germ-band elongated embryos, where strong expression appears first in PS13 and 14 and weaker in PS12 (compare panels A and F), followed slightly later by weaker expression in PS11 (compare panels B and G) and a barely detectable expression in PS10 (compare panela C and H). Panel D and E illustrate the resemblance between the *lacZ* (panel F) and *Abd-B* expression patterns (panel E) in stages 12 embryos. While the resemblance is strong in the epidermis and the central nervous system (CNS) of PS10 to PS13, panel D also reveals substantial ectopic expression in the CNS of the anterior parasegments. Signs of ectopic expression already appear earlier in extended germ band embryos as indicated by arrows in panel B,C and D Based on expression studies with the GFP reporter at later stages, we believe that the regions of ectopic expression indicated by the arrows in B, C and D) will give rise to the salivary gland. At present, we do not know if this ectopic expression (in the CNS and the salivary glands) results from a position effect of the genomic sequences in the vicinity of the insertion site of the BAC, or whether it reflects a normal aspect of endogenous BX-C regulation (where *Abd-B* might be repressed by another, potentially post-transcriptional mechanism). We note that we do not detect this ectopic expression when antibodies directed against *Abd-B* are used in embryos carrying the *Abd-B* BAC derivative (see above). Furthermore and as mentioned above, we never observe GOF phenotypes reminiscent of ectopic *Abd-B* expression in anterior segments. As the BAC constructs are identical outside of the insertion of the GAL4 coding sequences and are inserted into the same genomic location, we believe this enhanced ectopic expression in the GAL4 expressing BAC results from the amplification of a weak primary signal through the expression of a strong, secondary transcription factor (GAL4).

Supplementary Materials and Methods:

**Generation of Abd-B RNAi flies**. For easier visualization of the secondary cells, we first recombined the D5-Gal4 containing chromosome with a UAS-GFP line on the second chromosome to create the fly line “D5-Gal4, UAS-GFP”. A fly line carrying an Abd-B-specific RNAi construct (transformant ID 12025) was obtained from the VDRC (Vienna, Austria; [[89](#_ENREF_89)]). This UAS-AbdB^RNAi^ line was crossed to our D5-Gal4, UAS-GFP line driving its expression specifically in the secondary cells of the accessory glands. Male progeny positive for the D5-Gal4, UAS-GFP and the UASAbdB^RNAi^ were dissected in PBS and their accessory glands imaged under a Zeiss fluorescent microscope using standard methods. In order to improve the RNAi knockdown, we also recombined the D5-Gal4, UAS-GFP chromosome with a UAS-DICER2 chromosome (VDRC Stock 60008), also obtained from the VDRC. D5-Gal4, UAS-GFP, UAS-DICER males were used for the phenocopy experiments using the same protocols mentioned in the main manuscript.

The UAS lines (“UAS-AcpRNAi”) that produce dsRNA for CG1656, CG1652, and CG17575 are described in [37]. The UAS-AcpRNAi line for knockdown of Acp62F is from the VDRC [90] We used the ubiquitous driver *tubulin*-GAL4/TM3, Sb to verify the effectiveness of the knockdown by each UAS-AcpRNAi and the secondary cell driver *iab-6*D1-GAL4/CyO to check for secondary cell based knockdown. Each homozygous UAS-AcpRNAi line was crossed to each driver, and the balancer siblings from each cross (UAS-AcpRNAi/TM3, Sb for *tubulin* and UAS-AcpRNAi/CyO for *iab-6*D1) were used to control for background and environmental effects. Each lane contains extracts from two pairs of male accessory glands from virgin 3-5 day old males. The blots were probed for the protein that corresponded with the UAS-AcpRNAi line to check for knockdown; the other proteins were probed as loading controls. The western blots and samples were generated as described in the main manuscript.
